# PICK1 links Argonaute 2 to endosomes in neuronal dendrites and regulates miRNA activity

**DOI:** 10.1002/embr.201337631

**Published:** 2014-04-10

**Authors:** Anna Antoniou, Marcio Baptista, Nicholas Carney, Jonathan G Hanley

**Affiliations:** School of Biochemistry, University of BristolBristol, UK

**Keywords:** RISC, synaptic plasticity, trafficking

## Abstract

MicroRNAs fine-tune gene expression by inhibiting the translation of mRNA targets. Argonaute (Ago) proteins are critical mediators of microRNA-induced post-transcriptional silencing and have been shown to associate with endosomal compartments, but the molecular mechanisms that underlie this process are unclear, especially in neurons. Here, we report a novel interaction between Ago2 and the BAR-domain protein, PICK1. We show that PICK1 promotes Ago2 localization at endosomal compartments in neuronal dendrites and inhibits Ago2 function in translational repression following neuronal stimulation. We propose that PICK1 provides a link between activity-dependent endosomal trafficking and local regulation of translation in neurons.

## Introduction

MicroRNAs (miRNAs) are small RNA species encoded in the genome that associate with Argonaute proteins in the RNA-induced silencing complex (RISC) and mediate post-transcriptional silencing of one or more messenger RNA (mRNA) targets 1, 2. Neuronal-specific miRNAs drive development and neuronal identity 3 and have important implications in various brain disorders 4. Notably, a number of dendritic miRNAs affect spine morphology by targeting actin-regulating pathways 5 and regulate memory and synaptic plasticity 6, 7. Argonaute has been shown to localize to the post-synaptic density (PSD), suggesting a role in synaptic regulation 8. Interestingly, a fraction of Argonaute 2 (Ago2) associates with late endosomes and multivesicular bodies (MVBs) in non-neuronal cells 9, 10 where it is thought to participate in miRNA loading cycles, by associating with Dicer and other components of the RISC 11.

In neurons, recycling endosomes are found within or at the base of dendritic spines where they are crucial for the delivery of additional membrane to allow spine growth and for the regulation of synaptic AMPA receptor number during synaptic plasticity 12, 13. PICK1 is a BAR- and PDZ-domain protein that associates with early and recycling endosomes 14–16 and regulates the trafficking of AMPA receptors in long-term depression (LTD) and long-term potentiation (LTP) 16, 17. In addition, PICK1 regulates spine size by inhibiting Arp2/3-mediated actin polymerization 18, 19.

Here, we define a novel interaction between PICK1 and Ago2 in neurons and uncover a role for PICK1 in stabilizing Ago2 localization at endosomal compartments. We show that PICK1 affects Ago2 function by acting as an inhibitor of miRNA-mediated translational repression. Furthermore, we show that the interaction decreases following NMDA receptor stimulation (chemical LTD) and that PICK1 is involved in NMDA-induced changes in translational repression. This work therefore defines a novel mechanism for Ago2 regulation in neuronal dendrites, which plays a role in linking miRNA activity and synaptic plasticity.

## Results and Discussion

### Ago2 interacts with PICK1

**Figure 1:**
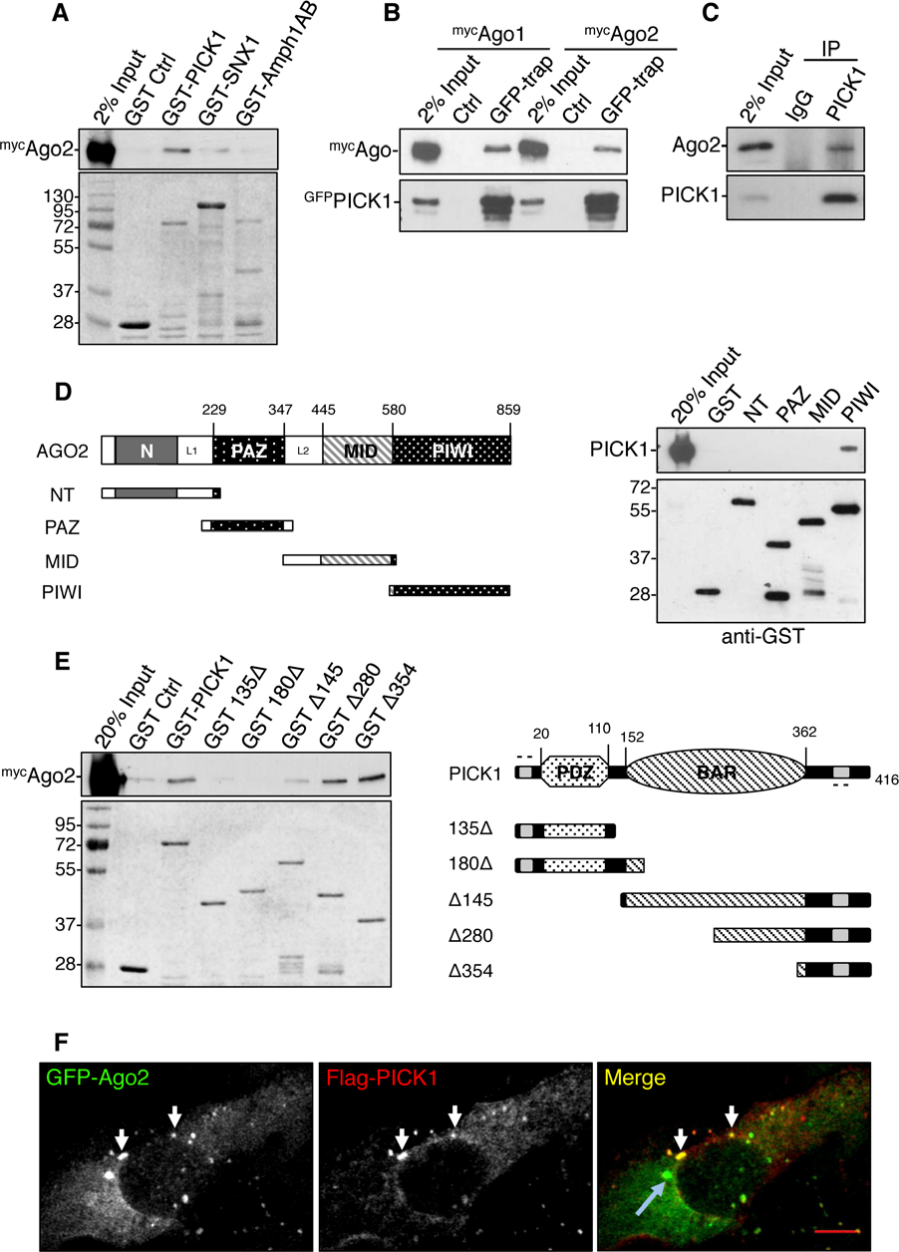
PICK1 interacts with Ago2 A Ago2 binds to GST-PICK1 but not other BAR-domain proteins tested. GST-PICK1, GST-SNX1 (sorting nexin 1), and GST-Amph1AB (Amphiphysin1 domains AB) were incubated with HEK293 cell lysate expressing ^myc^Ago2. Bound proteins were detected by western blotting using anti-myc or by Coomassie staining. B GFP-PICK1 interacts with both ^myc^Ago1 and ^myc^Ago2 from HEK293 cells. Lysates were incubated with GFP-trap agarose or blocked agarose beads as control, and bound proteins were detected by western blotting using anti-myc or anti-GFP. C Ago2 interacts with PICK1 in neurons. Lysates were immunoprecipitated with anti-PICK1 or control IgG, and bound proteins were detected by western blotting. D PICK1 interacts with the PIWI domain of Ago2. GST fusions of truncation mutants for the N-terminus (GST-NT) or the PAZ, MID, or PIWI domains as depicted in the diagram were incubated with purified his_6_-PICK1. Bound protein was detected by western blotting. E Ago2 interacts with the C-terminus of PICK1. GST-PICK1 full-length or truncation mutants as depicted in the diagram were incubated with HEK293 cell lysate expressing ^myc^Ago2. Bound proteins were detected by western blotting with anti-myc or by Coomassie staining. F PICK1 colocalizes with Ago2 puncta distinct from P-bodies. COS7 cells expressing GFP-Ago2 and flag-PICK1 were stained with anti-flag. White arrows indicate overlapping puncta between Ago2 and PICK1; blue arrow indicates a P-body; scale bar, 10 μm.

**Figure 2:**
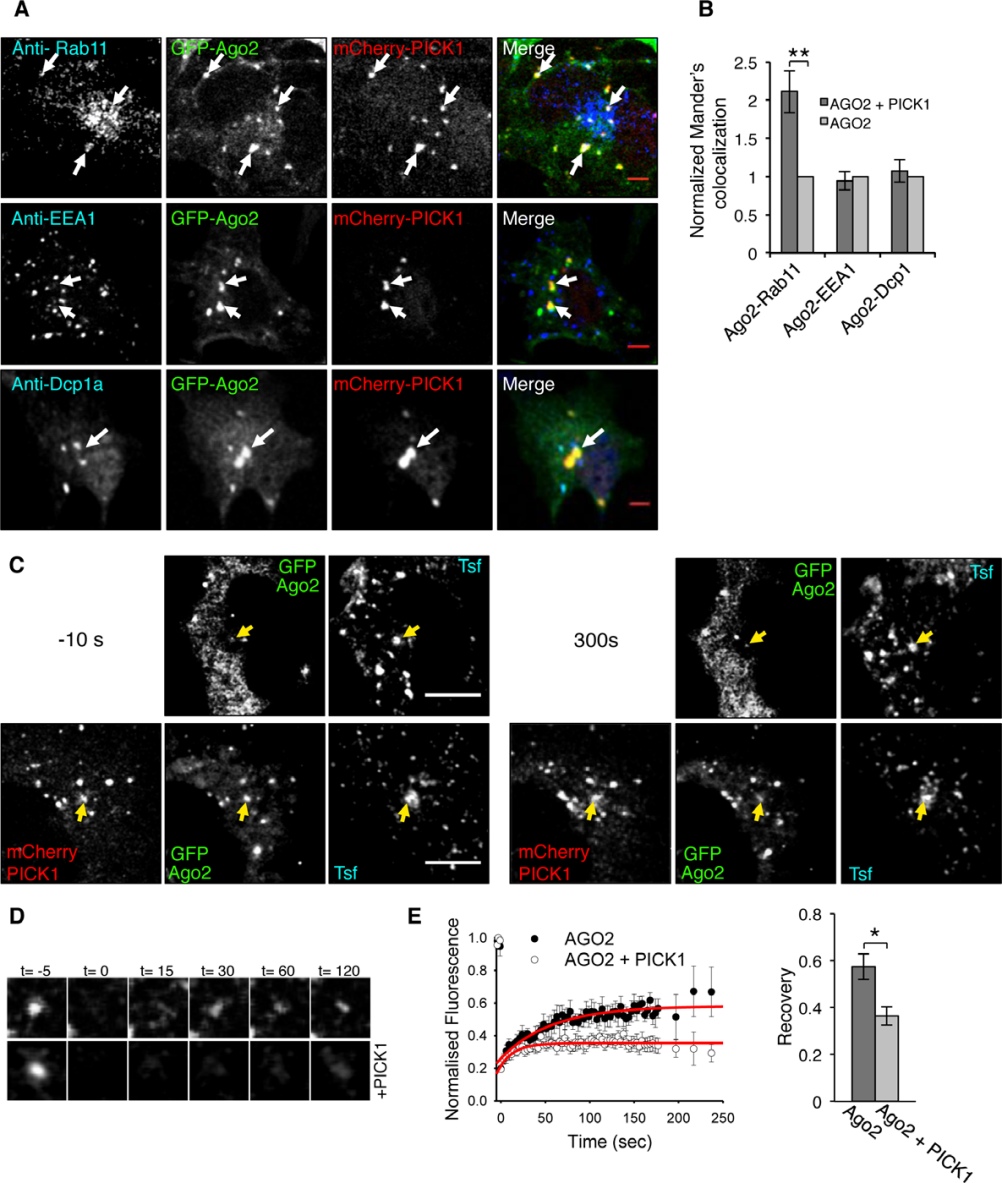
PICK1 promotes Ago2 association with recycling endosomes A PICK1 and Ago2 colocalize in Rab11-positive endosomal compartments in COS7 cells. Cells expressing GFP-Ago2 and mCherry-PICK1 were stained using specific antibodies against Rab11, EEA1, or Dcp1a (blue channel). Arrows indicate Ago2 and PICK1 overlapping puncta; scale bar, 5 μm. B PICK1 co-expression causes an increase in colocalization between Ago2 and Rab11. Colocalization analysis between Ago2 and Rab11, EEA1 or Dcp1a, was performed in COS7 cells expressing either GFP-Ago2 and mCherry-PICK1 or GFP-Ago2 alone. Graph shows Mander’s colocalization coefficients for the fraction of Ago2 colocalized with Rab11, EEA1, or Dcp1a in cells expressing both GFP-Ago2 and mCherry-PICK1 normalized to cells expressing Ago2 alone. **P = 0.0002 (Student’s *t*-test), *n* > 30 cells per condition. C Fluorescence recovery after photobleaching (FRAP) of GFP-Ago2 puncta colocalizing with transferrin-Alexa 647 (Tfn) in the absence or presence of mCherry-PICK1 in COS7 cells; scale bar, 10 μm. Images show cells pre-bleach (−10 s, left panels) and post-recovery (300 s, right panels). Yellow arrows indicate analyzed puncta. D Representative images of zoomed GFP-Ago2. Time (*t*) is in seconds; bleaching at *t* = 0. E Quantification of FRAP in (C) shows a decrease in recovery of Ago2 in the presence of PICK1. Fluorescence intensity was normalized to pre-bleach values, and fitted curves were used to extract recovery values. *P = 0.04 (Student’s *t*-test), *n* = 6–7 cells per condition.

In a search for novel PICK1 interactors, we conducted a proteomics screen following GST-pulldowns using GST-PICK1 as bait. One of the positive interactors was identified as Ago2. We verified the interaction by GST pulldown and found that Ago2 interacts with PICK1 but not with two other N-BAR-domain proteins, sorting nexin 1 (GST-SNX1) and Amphiphysin 1 (GST-Amph1AB) 20 (Fig 1A). We confirmed the interaction by co-precipitation from HEK293 cells, demonstrating an interaction between GFP-PICK1 and both myc-Ago2 and myc-Ago1 (Fig 1B). As PICK1 function has been extensively studied in neurons, we carried out coimmunoprecipitations from cultured neurons, which demonstrates that endogenous Ago2 and PICK1 interact in these cells (Fig 1C). To investigate the binding domains between Ago2 and PICK1, we performed pulldowns with GST-Ago2 truncation mutants. We found that PICK1 binds specifically to the PIWI domain of Ago2 (Fig 1D). Interestingly, Dicer and GW182 also bind the same region of Ago2 21, 22. In addition, we show that the C-terminal tail of PICK1, GST-Δ354, is sufficient for binding Ago2 (Fig 1E). To investigate the subcellular localization of the PICK1–Ago2 interaction, we expressed both proteins in COS7 cells. We found that flag-PICK1 and GFP-Ago2 form overlapping puncta (Fig 1F), which are distinct from RNA processing bodies identified by Dcp1a immunostaining (Fig 2A).

### PICK1 promotes the association of Ago2 with endosomal compartments

As PICK1 associates with endosomes and regulates endosomal trafficking 14–16 and Ago2 also associates with intracellular membranes 9, 10, 23, 24, we investigated the colocalization of Ago2 with PICK1 at endosomal compartments. We co-expressed GFP-Ago2 and flag-PICK1 in COS7 cells and co-stained for endosomal markers EEA1 or Rab11. We detected PICK1–Ago2 colocalization at Rab11-positive recycling endosomes, but not at EEA1-positive early endosomes (Fig 2A). To explore the role of the PICK1–Ago2 interaction, we asked whether PICK1 is involved in regulating the association of Ago2 with endosomes. We quantified colocalization between GFP-Ago2 and EEA1, Rab11, or the P-body marker Dcp1a, in cells transfected with both GFP-Ago2 and mCherry-PICK1 and cells expressing GFP-Ago2 alone. We found that PICK1 dramatically enhances the colocalization of Ago2 with Rab11, but not with EEA1 or Dcp1a (Fig 2B). To investigate the role of PICK1 in the dynamic localization of Ago2 at endosomes, we performed fluorescence recovery after photobleaching (FRAP) in COS7 cells expressing GFP-Ago2 with or without mCherry-PICK1, incubated with labeled transferrin to identify endosomes. We selected overlapping puncta, bleached GFP-Ago2, and quantified the recovery of fluorescence. Figure 2C–E shows that the presence of PICK1 reduces the recovery of GFP-Ago2 fluorescence at transferrin-positive compartments, suggesting that PICK1 either slows the recruitment of GFP-Ago2 to endosomes or that PICK1 stabilizes the association of Ago2 with endosomes. Taken together, these data suggest that PICK1 regulates the endosomal pool of Ago2, most likely by stabilizing Ago2–endosome interactions.

**Figure 3:**
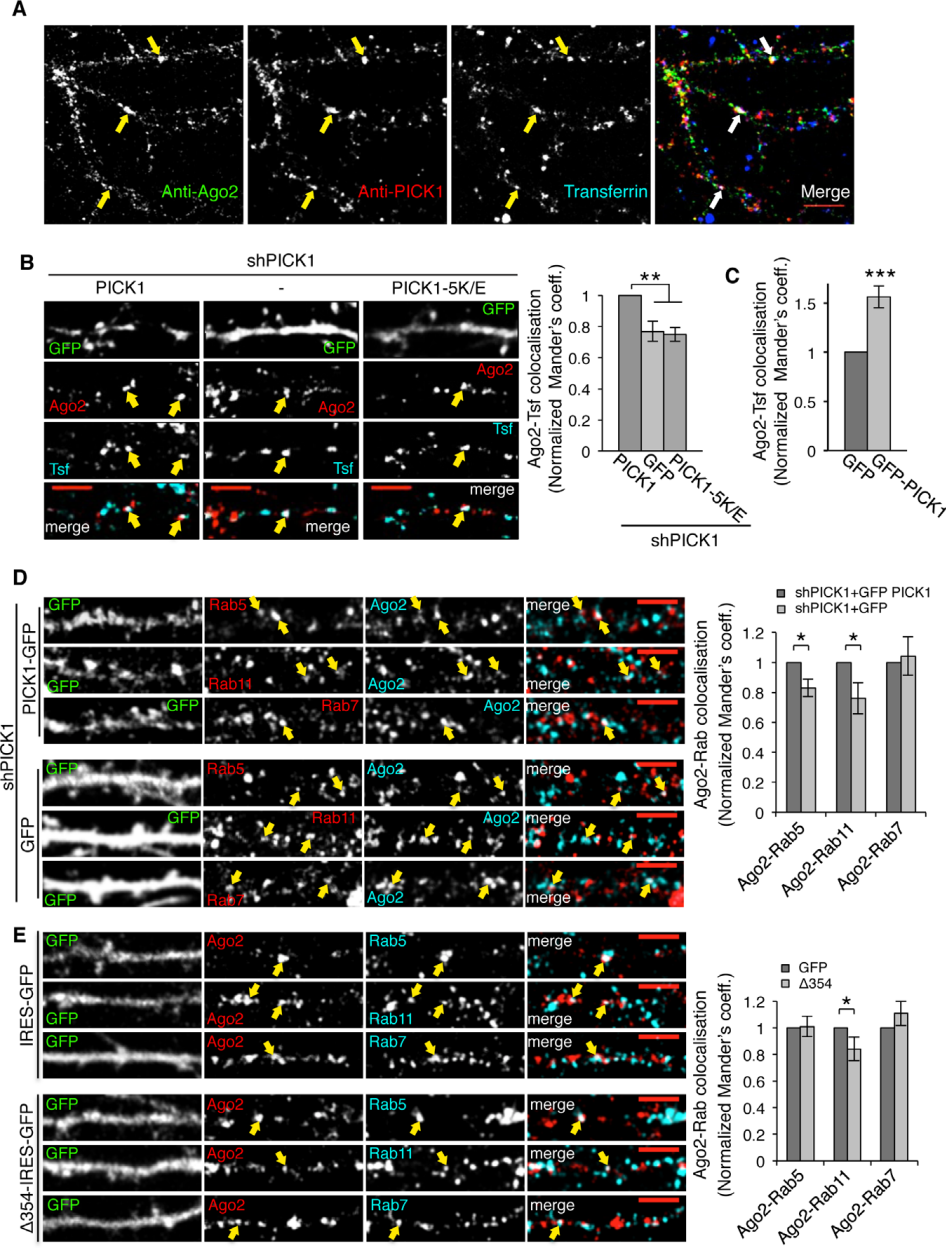
PICK1 promotes Ago2 localization at endosomal compartments in dendrites of hippocampal neurons A Endogenous Ago2 and PICK1 are found at transferrin-positive compartments in neuronal dendrites. Neurons were incubated with Alexa-conjugated transferrin to label endosomes and stained with Ago2 and PICK1 antibodies. Arrows indicate colocalizing puncta; scale bar, 10 μm. B PICK1 knockdown reduces Ago2 colocalization with endosomes. Neurons expressing shPICK1 plus GFP, sh-resistant GFP-PICK WT, or sh-resistant GFP-PICK 5K/E mutant were incubated with Alexa-conjugated transferrin (Tfn) and stained for Ago2. Bottom panels show the merge of Ago2 and Tfn channels. Scale bars, 5 μm. Graph shows Mander’s coefficients for the fraction of Ago2 colocalized with Tfn, normalized to GFP-PICK1 WT rescue condition. Arrows indicate overlapping puncta positive for Ago2 and transferrin. **P < 0.005 (Student’s *t*-test with Bonferroni correction), *n* > 10 cells per condition. C PICK1 overexpression increases Ago2 colocalization with endosomes. Cells expressing GFP or GFP-PICK1 were processed and analyzed as in (B). Representative images are shown in Supplementary Fig S2A. Graph shows Mander’s colocalization coefficients for the fraction of Ago2 colocalized with Tfn, normalized to GFP condition. ***P < 0.001, *n* > 10 cells per condition. D PICK1 knockdown reduces Ago2 colocalization with Rab5 and Rab11. Neurons expressing shPICK1 plus GFP or sh-resistant GFP-PICK1 were stained for Ago2 (cyan) and Rab5, Rab11, or Rab7 (red). Far right panels show the merge of Ago2 and Rab channels. Arrows indicate overlapping puncta positive for Ago2 and Rab protein; scale bars, 5 μm. Graph shows Mander’s coefficients for the fraction of Ago2 colocalized with Rab protein, normalized to GFP-PICK1 wild-type rescue condition. *n* > 8 cells per condition, *P < 0.05 (Student’s *t*-test), **P < 0.01. E Δ354 expression reduces Ago2 colocalization with Rab11. Neurons expressing empty-IRES-GFP or Δ354-IRES-EGFP were processed as in (D). Arrows indicate overlapping puncta positive for Ago2 and Rab protein. Graph shows Mander’s coefficients for the fraction of Ago2 colocalized with Rab protein, normalized to GFP control. *P = 0.03 (Student’s *t*-test), *n* > 9 cells per condition. Source data are available online for this figure.

To explore the localization of Ago2 and PICK1 at endosomes in neurons, we initially stained for PICK1 and Ago2 after incubating hippocampal neurons with transferrin. We found that a proportion of Ago2 colocalizes with transferrin-positive compartments in distal dendrites of hippocampal neurons and that PICK1 associates with a subset of these compartments (Fig 3A). To investigate the role of PICK1 in Ago2 localization, we transfected hippocampal neurons with plasmids expressing PICK1 shRNA and either GFP or shRNA-resistant GFP-PICK1 wild type or GFP-PICK1 (5K/E) (Supplementary Fig S1) and quantified the colocalization of Ago2 with transferrin (Fig 3B). PICK1 (5K/E) is a previously characterized mutation that disrupts binding to lipid membranes and is required for PICK1 localization and function 25. To control for the possibility that shRNA expression *per se* affects Ago2 localization 26, we normalized values to the wild-type rescue condition. Figure 3B shows that PICK1 knockdown or replacement with GFP-PICK1 5K/E causes a decrease in colocalization between Ago2 and transferrin compared to rescue with GFP-PICK1 wild type. Furthermore, overexpression of GFP-PICK1 leads to an increase in colocalization of Ago2 with transferrin-stained compartments when compared to GFP control (Fig 3C and Supplementary Figs S1 and S2A).

To further define the endosomal compartments involved, we exploited the well-characterized segregation of Rab proteins to specific endosomal compartments and used Rab5, Rab11, and Rab7-specific antibodies to label early, recycling, and late endosomes, respectively 27. Figure 3D shows that Ago2 partially colocalizes with all three of these endosomal compartments in neuronal dendrites. PICK1 knockdown causes a reduction in Ago2 colocalization with Rab5 and with Rab11, but has no effect on Ago2 colocalization with Rab7 (Fig 3D). To disrupt Ago2–PICK1 interactions while maintaining endogenous expression levels of both proteins, we expressed Δ354 PICK1 to compete with endogenous PICK1 for Ago2 binding. Although the Arp2/3 complex is a previously reported interactor with the PICK1 C-terminus, PICK1 Δ354 is insufficient to support Arp2/3 binding 18; therefore, this interaction would be unaffected in these experiments. Neurons expressing Δ354 PICK1 exhibit a reduced colocalization between Ago2 and the recycling endosomal marker Rab11, and no effect on colocalization with Rab5 or Rab7 (Fig 3E). Taken together, these results demonstrate that PICK1 regulates the association of Ago2 with recycling endosomes but not late endosomes in neuronal dendrites and that this requires the lipid-binding property of the BAR domain.

### PICK1 regulates miRNA-mediated translational repression

**Figure 4:**
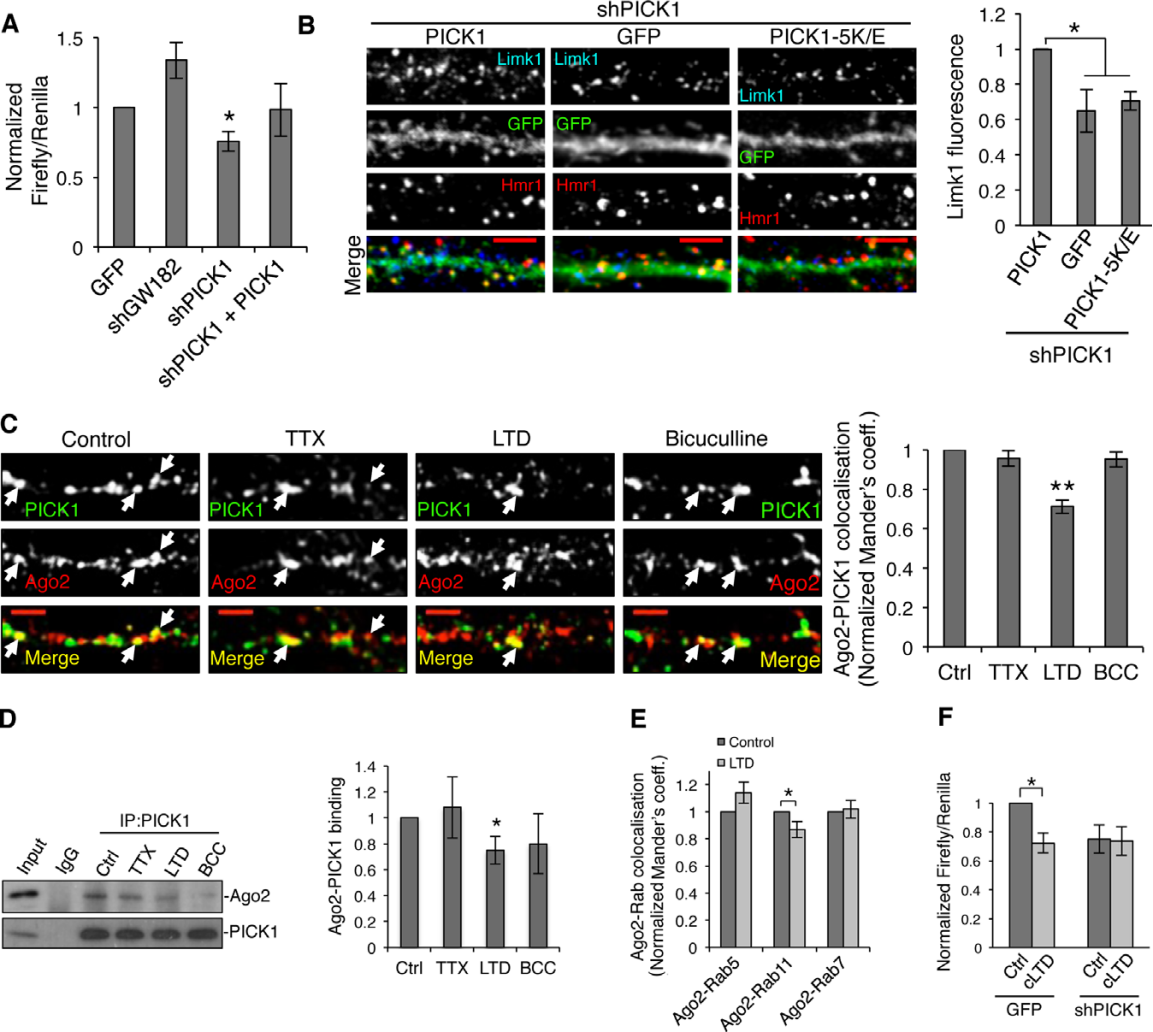
PICK1 is involved in microRNA-mediated silencing pathways during chemical LTD A PICK1 knockdown causes an increase in translational repression in cultured neurons. Dual-luciferase assay was performed in neurons expressing Renilla and Firefly luciferase reporter containing Limk1 3′UTR and either GFP, shGW182, shPICK1, or shPICK1 + sh-resistant GFP-PICK1. Values were normalized to the GFP-alone condition. *P < 0.05 (Student’s *t*-test with Bonferroni correction), *n* = 4–7. B PICK1 knockdown causes a decrease in endogenous Limk1 expression in neuronal dendrites. Neurons expressing shPICK1 plus GFP, sh-resistant GFP-PICK wild-type, or sh-resistant GFP-PICK 5K/E mutant were stained for Limk1 (blue) and the synaptic marker Homer1 (red). Scale bars, 5 μm. Graph shows Limk1 immunofluorescence normalized to GFP-PICK1 wild-type rescue condition. *P < 0.05 (Student’s *t*-test with Bonferroni correction), *n* > 14 cells per condition. C Chemical LTD causes a decrease in colocalization between Ago2 and PICK1 in dendrites of hippocampal neurons. Hippocampal neurons were treated with tetrodotoxin (TTX), Bicuculline (BCC), or NMDA (LTD), as shown and stained for PICK1 (green) and Ago2 (red). Arrows indicate overlapping puncta positive for PICK1 and Ago2. Scale bars, 5 μm. Graph shows Mander’s coefficients for the fraction of Ago2 colocalized with PICK1, normalized to untreated controls. **P < 0.01 (Student’s *t*-test with Bonferroni correction), *n* > 16 cells per condition. D Chemical LTD reduces the Ago2–PICK1 interaction. Neuronal cultures were treated as in (C), lysates were immunoprecipitated with anti-PICK1 or control IgG and bound proteins detected by western blotting. Values for Ago2 were normalized to PICK1 IP and to untreated control. *P < 0.05 (Student’s *t*-test with Bonferroni correction), *n* = 5–7. E Chemical LTD reduces the colocalization between Ago2 and Rab11. Neurons were treated for chemical LTD and stained with Ago2 and Rab proteins. Representative images are shown in Supplementary Fig S2B. Graph shows Mander’s coefficients for the fraction of Ago2 colocalized with Rab protein, normalized to untreated controls. *P = 0.03 (Student’s *t*-test), *n* > 9 cells per condition. F PICK1 knockdown occludes the effect of chemical LTD on translational repression. Dual-luciferase assay was performed in cultured neurons expressing GFP or GFP + shPICK1 and luciferase constructs as in (C). Neurons were treated for chemical LTD and lysed after 5 min. Relative luciferase values were normalized to vehicle-treated GFP control. *P = 0.03 (Student’s *t*-test), *n* = 8. Source data are available online for this figure.

Our data so far demonstrate that PICK1 regulates Ago2 localization, so we next investigated whether PICK1 is involved in miRNA-mediated translational repression. We determined the effect of PICK1 knockdown in cortical neurons in dual-luciferase assay using Renilla control and Firefly *Limk1* 3′UTR reporter, which is targeted by endogenous miRNA-134 28. We observed a decrease in relative luciferase value in PICK1 knockdown compared to GFP controls, an effect that is rescued by shRNA-resistant GFP-PICK1 (Fig 4A). This indicates that PICK1 knockdown enhances miRNA activity, suggesting that PICK1 inhibits miRNA-mediated translational repression under basal conditions. Moreover, these results suggest that PICK1 could modulate the translational repression of endogenous Limk1. To test this hypothesis, we analyzed Limk1 expression levels in dendrites of neurons expressing PICK1 shRNA. Neurons with reduced PICK1 expression exhibit a significant reduction in dendritic Limk1 expression (Fig 4B). Molecular replacement with PICK1 5K/E also causes a reduction in Limk1 expression, indicating that association with endosomal membranes is required for inhibition of Limk1 translational repression by PICK1.

### NMDA receptor activation modulates the PICK1–Ago2 interaction and translational repression

As PICK1 plays a critical role in synaptic plasticity and is well characterized for its role in LTD 17, we explored the effects of neuronal stimulation on the association of PICK1 and Ago2 in neurons. While global up- or down-regulation of synaptic activity by Bicuculline or TTX application, respectively, has no effect, chemical (c)LTD by bath application of NMDA causes a significant decrease in colocalization between PICK1 and Ago2 in neuronal dendrites (Fig 4C) and a decrease in the interaction by co-IP (Fig 4D). These results indicate that cLTD induction causes PICK1 and Ago2 to dissociate, suggesting that Ago2 dissociates from recycling endosomes. To test this, we analyzed Ago2 colocalization with Rab5-, Rab11-, and Rab7-positive compartments following cLTD induction. In agreement with our hypothesis, cLTD causes a small but significant reduction in colocalization between Ago2 and the recycling endosomal marker Rab11 and has no effect on the colocalization of Ago2 with Rab5 or Rab7 (Fig 4E and Supplementary Fig S2B).

We then explored the effect of PICK1 knockdown on miRNA-mediated silencing during cLTD, in dual-luciferase assays. We transfected cortical neurons with Firefly luciferase *Limk1* 3′UTR reporter and co-expressed either GFP control or PICK1 shRNA. Figure 4F shows that cLTD stimulation in GFP-expressing neurons causes a decrease in relative luciferase values, suggesting an up-regulation of miRNA-mediated silencing. This is in agreement with a previous study suggesting an up-regulation of miRNA expression in both LTP and LTD 29. The cLTD-induced up-regulation of miRNA-mediated silencing is occluded by PICK1 knockdown (Fig 4F), suggesting that PICK1 functions upstream of miRNA-mediated translational repression during cLTD.

As recycling endosomes are closely associated with dendritic spines, our results suggest that PICK1 recruits a population of Ago2 close to synapses, while inhibiting Ago2 function. Following NMDA receptor activation, the inhibition of Ago2 by PICK1 is removed, allowing an increase in miRNA-mediated translational repression. At the same time, Ago2 dissociates from recycling endosomes, translocating to an as yet undefined subcellular compartment, which may represent a site of Ago2-mediated translational repression in dendrites. However, the small proportion of Ago2 that we observe dissociating from endosomes suggests that a specific subpopulation of Ago2 may be regulated in this way. In conclusion, the PICK1–Ago2 interaction may function to link rapid changes in synaptic activity to longer-term changes in synaptic structure and function via local control of mRNA translation.

## Materials and Methods

### RNA interference

Knockdown and rescue constructs for shRNA PICK1 were gifts from Prof R. Malenka and were previously characterized 30. The constructs express H1-driven PICK1 shRNA and ubiquitin promoter-driven GFP or sh-resistant GFP-PICK1. For GW182 knockdown, we used shRNA against mouse TNRC6C in pSuper vector, which was a kind gift from Prof. G. Schratt.

### Cell treatments

Mature cortical or hippocampal cultures were treated with either 0.5 μM tetrodotoxin (TTX) for 1 h or 20 μM Bicuculline for 1 h. For chemical LTD, cultures were incubated with 0.5 μM TTX for 10 min and then stimulated with 20 μM NMDA (Tocris), 20 μM glycine, and 0.5 μM TTX for 3 min. Cells were used 5 min after stimulation. All treatments were performed in pre-warmed HEPES-buffered saline containing 140 mM NaCl, 5 mM KCl, 1.8 mM CaCl_2_, 0.8 mM MgCl_2_, 25 mM HEPES pH 7.4, and 10 mM glucose, pH 7.5.

### Co-immunoprecipitation

Immunoprecipitation was performed as previously described 18, 19. Briefly, cell extracts in lysis buffer were incubated with 2 μg of anti-PICK1 or control IgG antibodies and pulled down with protein G-sepharose beads (Sigma). Beads were washed in lysis buffer (0.5% Triton X-100, 150 mM NaCl, and 20 mM HEPES, pH 7.4). Bound proteins were detected by Western blotting with Ago2 or PICK1 antibodies. For quantification, Western blot films were scanned and analyzed in Image J (NIH). Two-tailed Student’s *t-*test was used to determine significance.

### Immunofluorescence and image analysis

Cells grown on coverslips were fixed in 4% paraformaldehyde (Thermo Scientific) in PBS (Sigma). Cells were permeabilized in 0.2% Triton-X100, 2% BSA for 10 min and incubated with primary antibodies in 5% bovine serum albumin (Sigma) for 1 h or overnight at 4°C followed by secondary antibodies for 45 min. Coverslips were imaged on a Zeiss LSM510 confocal system. Image processing and colocalization analysis were performed using ImageJ and JACoP 31. Three randomly selected 20 μM dendritic regions were analyzed per neuron. Mander’s fractions were measured using thresholding, and values were normalized for each of at least three independent experiments, and statistical significance was determined using two-tailed Student’s *t*-test.

### Fluorescence recovery after photobleaching

COS7 cells grown on coverslips were imaged at 37°C in HBS solution. Image conditions were optimized to minimize photobleaching induced by time-lapse imaging. Bleaching of GFP was achieved at 100% laser power at 488 nm for < 5 s and targeted to predefined circular regions of interest (ROI) of constant area. Five consecutive images were acquired pre-bleaching. Images were acquired post-bleaching at 3 s intervals, until a stable recovery plateau was reached. Quantification of recovery was performed in ImageJ. Sigma Plot was used to estimate half-life of recovery and visualize recovery curves.

### Luciferase assay

Dual-luciferase reporter assay system was purchased from Promega, and the assay was carried out following manufacturer’s instructions. Values were normalized for each of at least three independent experiments, and statistical significance was determined using two-tailed Student’s *t*-test.
