# Supplementary material for: PICK1 links Argonaute 2 to endosomes in neuronal dendrites and regulates miRNA activity
Source: EMBO Rep. 2014 Apr 10;15(5):548–56. doi: 10.1002/embr.201337631 (PMC4210090; doi:10.1002/embr.201337631)
Supplement: Supplementary file 1 [file embr0015-0548-sd1.pdf]

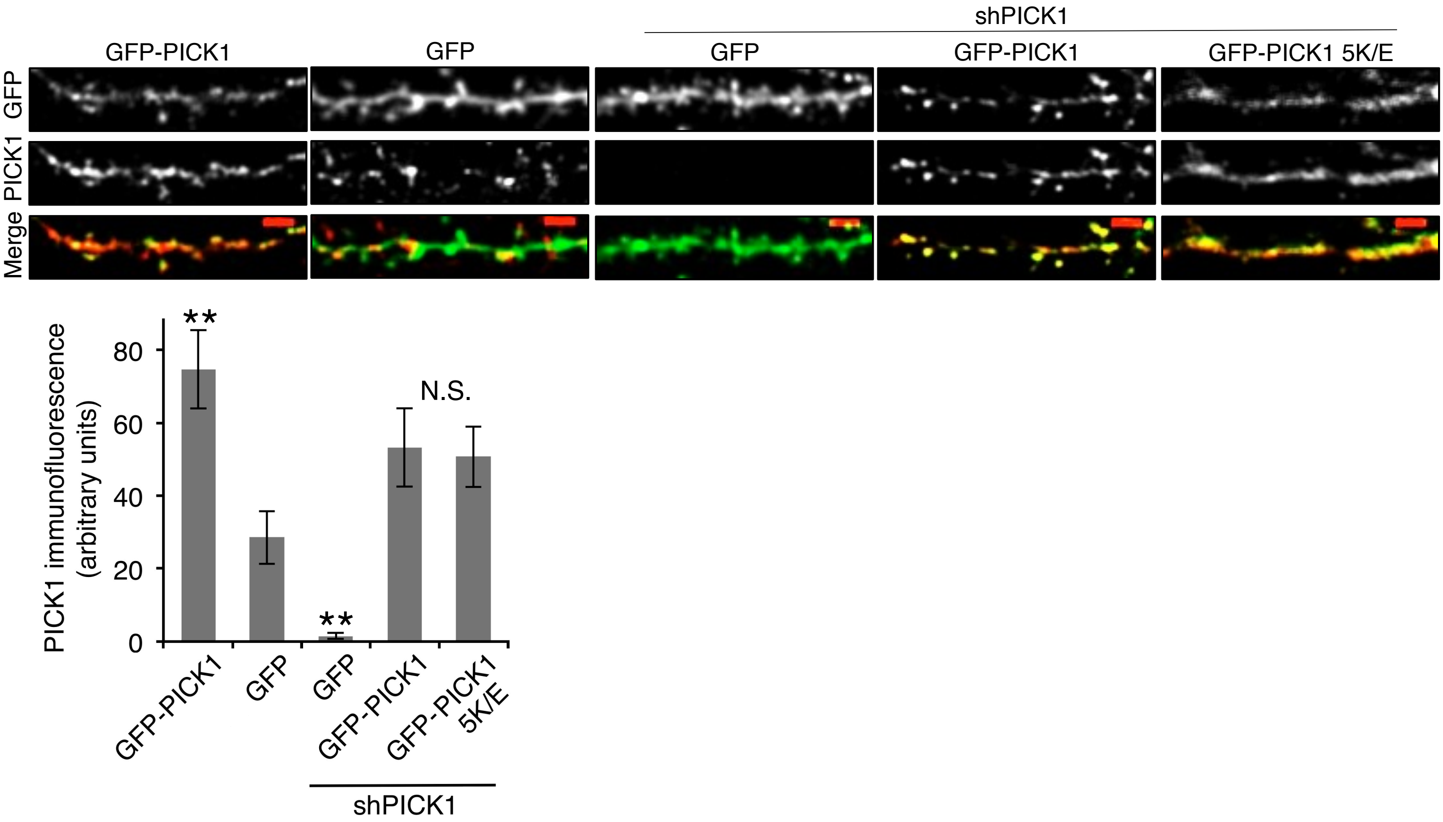

Supplementary Figure S1

Characterisation of PICK1 knockdown, rescue and overexpression.

Neurons expressing GFP, GFP-PICK1 or GFP-PICK1 5K/E with or without PICK1 shRNA as shown were stained for PICK1 (red channel). Graph shows mean PICK1 immunofluorescence intensity per cell. **$p<0.01$, relative to GFP control (t-test, with Bonferroni correction), n= 5 cells per condition.
