# Supplementary material for: PICK1 links Argonaute 2 to endosomes in neuronal dendrites and regulates miRNA activity
Source: EMBO Rep. 2014 Apr 10;15(5):548–56. doi: 10.1002/embr.201337631 (PMC4210090; doi:10.1002/embr.201337631)
Supplement: Supplementary file 2 [file embr0015-0548-sd2.pdf]

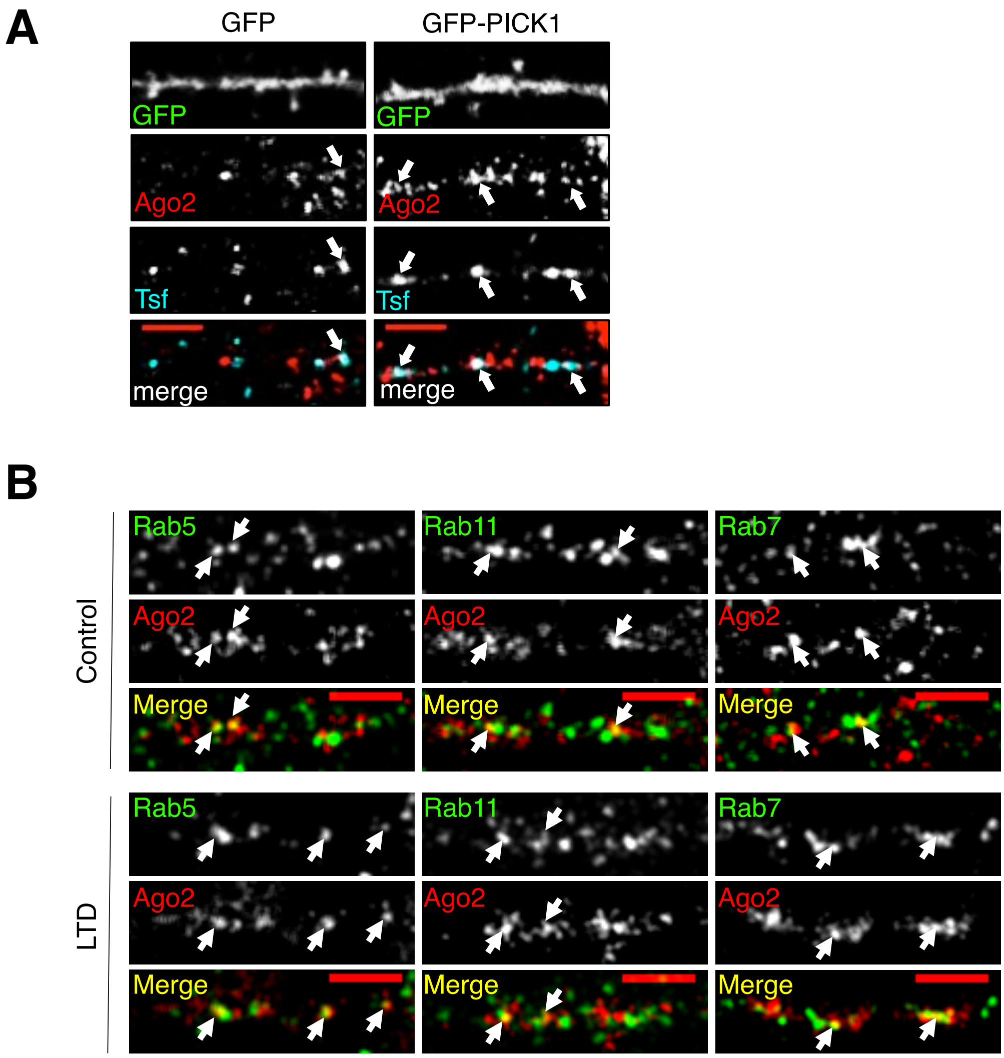

Supplementary Figure S2

A) Representative images for Figure 3C. Neurons expressing GFP or GFP-PICK1 were incubated with Alexa-conjugated transferrin (Tfn, cyan) and stained for Ago2 (red). Bottom panels show the merge of Ago2 and Tfn channels. Scale bars 5μm.

B) Representative images for Figure 4E. Neurons were treated for chemical LTD and stained with Ago2 (red) and Rab proteins (green). Scale bars 5μm.
