# Supplementary material for: PICK1 links Argonaute 2 to endosomes in neuronal dendrites and regulates miRNA activity
Source: EMBO Rep. 2014 Apr 10;15(5):548–56. doi: 10.1002/embr.201337631 (PMC4210090; doi:10.1002/embr.201337631)
Supplement: Supplementary file 3 [file embr0015-0548-sd3.pdf]

Supplementary Methods

Materials

Antibodies used in immunofluorescence were as follows; mouse monoclonal anti-Dcp1a (Abcam, ab57654), monoclonal mouse antibody against the N-terminus of Ago2 (Wako Chemicals), rabbit anti-PICK1 antibody (Abcam, ab3420), anti-Rab11 (BD Biosciences) and anti-EEA1 mouse (Sigma). anti-Rab5, Rab11, Rab7 rabbit (Cell Signaling). For biochemistry, mouse anti-PICK1 (NeuroMab) and goat N-terminal anti-PICK1 (Santa Cruz, sc-9539) were used in western blot and immunoprecipitation, respectively. Rabbit monoclonal anti-Argonaute 2 (Cell Signaling, 2897), mouse anti-Myc (Cell Signaling), anti-flag mouse (Sigma-Aldrich) and anti-GST (GE Healthcare) were used in western blot. Transferrin-Alexa Fluor647 or 633 was purchased from Invitrogen.

Plasmid preparation

Mammalian GST-bound Ago2 truncation mutants were amplified from human AGO2 in pEBG vector by PCR with the following pairs of oligonucleotides, with a *Bam*HI or *Not*I linker (underlined) or a stop codon (bold) :

5'-TATGGATCCAACATCCTGCTGCCC-3' (Met primer, sense) and
5'-ATAGCGGCCGC**TCA**AGCAAAGTACATGGTGC-3' (stop primer, anti-sense) for GST-PIWI;

5'-TATGGATCCCTGGAGGTCTGTAACATTGTG-3' (Met primer, sense) and
5'-ATAGCGGCCGC**TCA**CGGCCTGCCCTGGGG-3' (stop primer, anti-sense) for GST-MID;

5'-TATGGATCCGCACAGCCAGTAATCGAG-3' (Met primer, sense) and
5'-ATAGCGGCCGC**TCA**TTTAATACATCTTTGTCCTGC-3' (stop primer, anti-sense) for GST-PAZ.

The cDNAs obtained were subcloned into pEBG vector using KOD Hot start polymerase kit (Novagen). Full-length GST-Ago2 and truncation mutant GST-NT were kind donations from Tom Hobman, University of Alberta, USA [1].

Cell Culture and transfection

Primary neuronal cultures were prepared from E18 Wistar rats using standard procedures. Cells were plated on poly-L-lysine coated plates or coverslips in Neurobasal medium (Gibco), supplemented with 5% Horse Serum (Gibco), 2% B27 (Gibco), 1% Penicillin/Streptomycin (Sigma) and 2mM glutamine (Gibco). Plating media was replaced by Neurobasal medium excluding Horse Serum at least two hours after plating. Neurons were transfected with plasmid DNA at 10-13 days in vitro using Lipofectamine 2000 (Invitrogen) and used for experiments 4-5 days later. COS7 and HEK293T cells were transfected using JetPEI (Polyplus) or TransIT-LT1 (Mirus) transfection reagents.

GST-pulldown assay

Purified GST-bound proteins were prepared from BL21 bacterial cultures as previously described [2], or expressed as fusion proteins in HEK293T cells. Glutathione agarose beads (Sigma) were incubated with bacterial or mammalian cell lysates. They were then washed in lysis buffer (0.5% Triton X-100, 100mM NaCl and 20mM HEPES, pH7.4) or HTG buffer (150mM NaCl, 10% Glycerol, 25Mm HEPES, pH7.4) and incubated with either cleared HEK293T cell lysate or purified his_6-PICK1 in lysis buffer. After washing, bound proteins were analysed in western blotting.

Supplementary References

1. Tahbaz N, Carmichael JB, Hobman TC (2001) GERp95 belongs to a family of signal-transducing proteins and requires Hsp90 activity for stability and Golgi localization. *J Biol Chem* **276:**43294-43299

2. Rocca DL, Martin S, Jenkins EL, Hanley JG (2008) Inhibition of Arp2/3-mediated actin polymerization by PICK1 regulates neuronal morphology and AMPA receptor endocytosis. *Nat Cell Biol* **10:**259-271
