# Supplementary material for: PICK1 links Argonaute 2 to endosomes in neuronal dendrites and regulates miRNA activity
Source: EMBO Rep. 2014 Apr 10;15(5):548–56. doi: 10.1002/embr.201337631 (PMC4210090; doi:10.1002/embr.201337631)
Supplement: Supplementary file 4 [file embr0015-0548-sd4.pdf]

EMBO
reports

Manuscript EMBOR-2013-37631

PICK1 links Argonaute 2 to endosomes in neuronal dendrites and regulates microRNA activity

Anna Antoniou, Marcio Baptista, Nicholas Carney and Jonathan G. Hanley

Corresponding author: Jonathan G. Hanley, University of Bristol

Review timeline:

Submission date:	11 June 2013
Editorial Decision:	04 July 2013
Revision received:	20 December 2013
Editorial Decision:	10 January 2014
Revision received:	03 February 2014
Accepted:	25 February 2014

Transaction Report:

(Note: With the exception of the correction of typographical or spelling errors that could be a source of ambiguity, letters and reports are not edited. The original formatting of letters and referee reports may not be reflected in this compilation.)

Editor: Barbara Pauly

1st Editorial Decision 04 July 2013

Thank you very much for the submission of your research manuscript to our editorial office. We have now received the full set of reviews on your manuscript.

As the detailed reports are pasted below I will only repeat the main points here. You will see that the reviewers appreciate the interest of your findings and are, in principle, supportive of publication of your study in our journal. However, they also point out aspects of your study that would need to be further strengthened before publication. For instance, all referee agree that the effects of the PICK1-Ago interaction on the expression of endogenous target genes should be tested. Referee 1 and 2 also agree that the effects of interaction-deficient mutants on Ago localization and Ago-mediated gene suppression should be analyzed and referee 1 feels that the proposed translocation of Argonaute from the recycling to the late endosomes upon dissociation from PICK1 should be shown. Reviewer 3 remarks that the nature of the different endosomal compartments should be better defined and all reviewers point out instances in which additional controls are needed (for example for the interaction assays, the localization studies and the knockdown experiments).

Given these evaluations, the reviewers constructive comments and the potential interest of the study, I would like to give you the opportunity to revise your manuscript, with the understanding that the main concerns of the reviewers should be addressed. Acceptance of the manuscript will depend on a positive outcome of a second round of review and I should also remind you that it is EMBO reports policy to allow a single round of revision only and that therefore, acceptance or rejection of the

© European Molecular Biology Organization

manuscript will depend on the completeness of your responses included in the next, final version of the manuscript.

Revised manuscripts should be submitted within three months of a request for revision; they will otherwise be treated as new submissions. If you feel that this period is insufficient for a successful submission of your revised manuscript I can potentially extend this period slightly. Also, the length of the revised manuscript should not exceed roughly 29,000 characters (including spaces and references). If you feel that the additional data requested by the reviewers would make the manuscript too long you may consider including some peripheral data in the form of Supplementary information. However, Materials and Methods essential for the repetition of the main experiments should not be displayed as supplementary information only.

As part of the EMBO publication's Transparent Editorial Process, EMBO reports publishes online a Review Process File to accompany accepted manuscripts. This File will be published in conjunction with your paper and will include the referee reports, your point-by-point response and all pertinent correspondence relating to the manuscript.

You are able to opt out of this by letting the editorial office know (emboreports@embo.org). If you do opt out, the Review Process File link will point to the following statement: "No Review Process File is available with this article, as the authors have chosen not to make the review process public in this case."

We also welcome the submission of cover suggestions or motifs that might be used by our Graphics Illustrator in designing a cover.

I look forward to seeing a revised form of your manuscript when it is ready. Should you in the meantime have any questions, please do not hesitate to contact me.

REFEREE REPORTS:

Referee #1:

Antoniou et al report here on the association of the postsynaptic BAR domain protein PICK1, a regulator of glutamate receptor trafficking and the actin cytoskeleton with the argonaute 2 (Ago2), a key factor in miRNA-mediated post-transcriptional gene silencing. The authors show that Ago2 via its PIWI domain associates with the C-terminal region of PICK1 and both proteins partially colocalize on transferrin (Tf) positive endosomes (perhaps recycling endosomes, REs). KD of PICK1 reduces colocalization of Ago2 with Tf in neuronal dendrites and occludes chemical LTD induced miRNA-134 mediated gene silencing in luciferase reporter assays. The latter is consistent with the observation that cLTD reduces the binding of Ago2 to PICK1 in co-ips. Based on these data the authors speculate that cLTD via induction of Ago2 dissociation from PICK1 may facilitate Ago2 relocalization to late endosomes, where it drives miRNA-134 dependent translational repression.

The observation that PICK1 by directing Ago2 localization and function may regulate translational repression in neuronal dendrites is highly interesting. However, as it stands some of the data are preliminary and key aspects of the proposed model remain unproven. In particular it no data are provided regarding the hypothesized switch between PICK1-mediated recycling endosomal sequestration of inactive Ago2 and its relocation to late endosomes (LEs). Moreover, whether and how Ago2 complex formation indeed does regulate local translation of endogenous neuronal proteins has not been studied. Based on these concerns I cannot recommend publication of the Ms in EMBO Rep, at least in its present format.

Major points:
1. As stated above no data are presented regarding the proposed transition of Ago2 between inactive RE and LE pools. The authors need to carry out a thorough analysis of Ago2 localization at REs, EEs, LEs/ MVBs, and P-bodies under conditions of cLTD and in presence or absence of PICK1 as well as Ago2 binding defective mutants. Such data in my opinion are required to substantiate the

key conclusions of this work.

2. Equally important is the demonstration that complex formation between Ago2 and PICK1 indeed does regulate local translation(i.e. local amounts) of endogenous neuronal proteins.

3. The data shown in fig. 1 appear somewhat preliminary. For example, in fig. 1A it Ago2 also binds to GST-SNX1 (albeit perhaps more weakly); figs. 1B + 1C miss essential positive (i.e. the antigen itself) and negative controls. Moreover, it is difficult to judge the efficiency of the co-ips and pulldowns as no information is provided what fraction of the input material was loaded for comparison. Fig. 1D misses an input control altogether.

4. I am also unsatisfied with the quantification of colocalizations in Fig. 2. Normalized Pearson coefficents are hard to interpret and a much more extensive analysis with markers for LEs, MVBs, P-bodies etc. is required (see point #1 above).

5. The effect of KD of PICK1 on Ago2 localization to Tf-positive endosomes appears miniscule. Is this because of redundancy in the system? If so, why then does KD of PICK1 exhibit such profound effects on luciferase expression in the reporter assays? From the data it is possible that the effects of PICK1 on gene silencing are mediated by factors other than Ago2. Again, better and more direct data involving interaction-defective mutants, at least in dominant-negative assays are required to substantiate the hypothesis that PICK1 may regulate local translation via Ago2 sequestration. Further: Does KD of PICK1 indeed increase the localization of AGo2 with MVBs and P-bodies?

Referee #2:

Antoniou et al.

PICK1 links Argonaute 2 to endosomes in neuronal dendrites and regulates microRNA processing

In this manuscript, the authors have identified PICK1 as novel interactor of Ago2. Using a GST-PICK1 pull down they identify Ago2 as positive interactor. They verify the interaction in HEK293 cells and neurons using co-transfections and also endogenous proteins. In further experiments, interaction domains on both proteins were mapped. They also show that both proteins co-localize in granules that are distinct from P bodies. PICK1 regulates endosomal trafficking. Therefore, Antoniou et al. investigatde the interaction of both proteins at endosomal compartments and find that PICK1 enhances the association of Ago2 with such compartments. To further study the role of PICK1 in Ago2 localization in neurons, the authors performed PICK1 knock down and over expression studies. The knock downs were rescued by either wt of a lipid-binding deficient PICK1 variant. In these experiments, the authors find that Ago2 localization by PICK1 depends on the lipid-binding domain. Finally, they show that PICK1-Ago2 interaction is reduced upon NMDA receptor activation and PICK1 represses miRNA-guided gene silencing.

This is a short report on a potential role of PICK1 in miRNA-guided gene silencing in neurons. It is well written and the results are presented clearly. However, there are a number of points that need further validation or clarification.

1. The authors very often draw conclusions that are not supported by their data. For example, on page 4 (end of first results point) they end with '...suggesting that the pool of Ago2 associated with PICK1 is inactive.' This is not at all demonstrated in Figure 1 or 2. The observation that it does not co-localize with P bodies does not mean that Ago2 is in an inactive form. In fact, the role of P bodies in miRNA-guided gene silencing is rather elusive. This statement should be changed.

2. In the title, the authors claim that PICK1 regulates microRNA processing? I did not find a single experiment in this manuscript that would have tested this hypothesis.

3. The authors use shRNA-mediated knock down of PICK1. They should also present western blots showing knock down efficiency in their experiments. Especially neurons might be difficult to target and knock down efficiency should be validated.

4. In Figure 4B, the authors indicate by arrows Ago2-PICT1 co-localization upon treatment with different agents. They claim that LTD inhibits co-localization. Nevertheless, the images look quite similar to the others and also a white arrow indicates co-localization? This should be made clearer.

5. Figure 2D is not mentioned in the text.

6. A potential repressive effect of PICK1 on miRNA-guided gene silencing should be tested in more detail. Other proteins should be knocked down as additional controls since the effects are generally quite mild (this holds true for many of the presented experiments, but this might be due to the highly complex neuronal system). The effects on gene silencing are rescued by wt PICK1. An Ago2-binding deficient mutant should not rescue the phenotype. This could be tested. Does PICK1 binding prevent Ago2-GW182 interaction?

7. Ago-association with membranes has also been analyzed in a recent publication by Stalder et al. (EMBO Journal). This manuscript could be cited as well.

Referee #3:

The manuscript PICK1 Links Argonaute to Endosomes in Neuronal Dendrites and Regulates MiRNA Processing by Antoniou et al. provides evidence for an association of PICK1 with Argonaute2, and effects on Argonaute localization to endosomes and miRNA activity. PICK1 has been attributed roles in endocytic and Golgi trafficking and contains domains involved in deforming membranes. The authors demonstrate that PICK1 associates with Ago2, although these may not necessarily interact directly. Since PICK1 has been linked to endocytic trafficking the authors demonstrate that PICK1 increases co-localization of Ago2 with Rab11+ compartments, likely recycling endosomes. Since PICK1 is involved in long-term depression (LTD) the authors test in the latter part of the manuscript the effect of a chemical model of LTD on association and localization of PICK1 on Ago2. While the effects observed in this part of the paper are small they are reasonably supported by the evidence if responses to the comments below are satisfactory.

The manuscript is a novel contribution to our understanding of the cell biology of RNA silencing, and the dynamic control of miRNA activity in neuronal dendrites. This paper will be of some interest to the broad field of post-transcriptional regulation and neuroscience. While the principal findings and claims of the paper are interesting the paper lacks critical controls in several places and greater methodological detail is required to determine the value of other experiments.

MAJOR CONCERNS
1. In Fig. 1a the authors show blots of AGO2 co-purified with GST-tagged BAR domain proteins. Weak bands for AGO2 are visible in control lanes and SNX1 lanes. The authors should temper there conclusions on the specificity of PICK1 accordingly.
2. Fig. 1b several controls are missing for the authors to conclude that PICK1 interacts with AGO2 but not AGO1. First the authors need to demonstrate that they immunoprecipitated equivalent amounts of Flag-PICK1 in each lane. They also need to demonstrate that equivalent amounts of myc-AGO1 and myc-AGO2 were expressed in cells.
3. (a) The authors could build much more confidence in their experiments and reagents by confirming their specificity. This is particularly important since the authors switch between measuring Ago2 localization to Rab11, Tfn and PICK1 in different experiments. For example, the authors should demonstrate that overexpressed PICK1 localizes similarly to endogenous and should highlight data demonstrating that PICK1 labels endosomes (Tfn) and Rab11+ compartments by co-labeling. The authors should similarly confirm PICK1 antibody staining of endosomes.
(b) Staining pattern in Fig. 2A for Rab11 is unusual for recycling endosomes. To confirm the antibody is specific and these are putative recycling endosomes they should treat cells with Rab11A and B-specific siRNA and stain with anti-Rab11 antibody. The authors should show to what extent Rab11 and Tfn foci overlap as well since they often use Tfn as a surrogate marker of Rab11+ or PICK1-affected endosomes. Given that the authors rely on transferrin heavily in the rest of the paper to study AGO2 co-localization with endosomes they should repeat the experiments in Fig. 2A

testing AGO2 co-localization with Tfn.
4. For all experiments demonstrating quantification of imaging the authors should note in the figure legend or on the figure itself the number of cells or images analyzed, as well as an average Pearson's correlation coefficient for each condition. It would be helpful to readers to list on the y-axis which two markers were tested for co-localization.
5. On p.4 the authors state that since PICK1 co-localizes with AGO2 outside P-bodies, that this AGO2 must be in an inactive form. Literature demonstrates that miRNA function normally in the absence of P-bodies. (e.g. P-Body Formation Is a Consequence, Not the Cause, of RNA-Mediated Gene Silencing, Eulalio et al. 2007), therefore the authors' statement should be modified.
6. (a) In FRAP experiments in Fig. 2C-D the authors propose that they examine AGO2 recovery after photobleaching of a foci that overlaps with an Tfn+/PICK1+ foci (presumably an endosome). These experiments require complex controls. First, endosomes are highly dynamic so it is very unlikely that the Tfn+/PICK1+ structure remains stationary in the imaged region if it is an endosome in a healthy cell. Second, the z-plane of capture tends to drift in these experiments, so apparent differences in recovery are easily skewed by planes. The authors should show images over the FRAP period with a second marker of the GFP-AGO2 foci that is unaffected by the bleach and demonstrates that the foci is stationary (e.g. Tfn-Alexa647 or mCherry-PICK1 should be more photostable than GFP).
(b) The authors cannot conclude from their FRAP experiment that PICK1 stabilizes AGO2 at endosomes. They can only conclude that PICK1 slows recruitment of AGO2 to endosomes.
7. The authors frequently test the function of PICK1 by overexpression. The authors need to show western blots of endogenous PICK1 levels in cells vs. levels in cells with overexpressed PICK1. Similarly the authors need to show evidence by western blot of knockdown of PICK1 in Fig. 3-4 and rescue with PICK1 and mutated PICK1.
8.Given that many of the effects the authors observe are weak the statistical tests they applied are important. The authors should stipulate whether one- or two-tailed t-tests were applied, the type, and the p-value considered significant. At times ANOVA tests may be required to compare more than two samples.
9. The functional or physiological relevance of the paper could be significantly increased if the authors reinforced the data showing an effect on miRNA activity (NOT processing) by testing whether PICK1 affects levels of LIMK1 protein in neurons and affects dendritic spine morphology as suggested by the miR-134 vector that they use from Schratt GM et al. Nature 2006.
10. I would recommend changing the title from "MiRNA Processing" to "MiRNA Activity". MiRNA processing suggests that the observed effects are on pre-miRNA cleavage or other stages of miRNA biogenesis or modification.

MINOR POINTS
1. In Fig. 1b the IP is labeled as myc-AGO1 or -2 and the blot is labeled as myc-AGO. One of these is mislabeled.
2. The IP-western blot in Fig. 4a is not labeled with the identities of the proteins blotted.
3. Fig. 1E would suggest that the BAR domain inhibits binding of AGO2 to the PICK1 C-terminus. What might this imply for localization at endosomes because of known functions of the BAR domain? What if anything is known about the AGO2 binding site in the PICK1 C-terminus? The authors should comment.

1st Revision - authors' response 20 December 2013

Response to reviewers

We thank the reviewers for their constructive comments, which we have addressed point-by-point, below.

<u>Specific responses to Reviewer 1:</u>

1. As stated above no data are presented regarding the proposed transition of Ago2 between inactive RE and LE pools. The authors need to carry out a thorough analysis of Ago2 localization at REs, EEs, LEs/ MVBs, and P-bodies under conditions of cLTD and in

presence or absence of PICK1 as well as Ago2 binding defective mutants. Such data in my opinion are required to substantiate the key conclusions of this work.

We used a range of Rab-specific antibodies as markers for endosomal compartments (Rab5: early endosomes, Rab11: recycling endosomes, Rab7: late endosome-lysosomes). In colocalisation studies, we found that while Ago2 shows partial colocalisation with all three markers, PICK1 knockdown reduces Ago2 association with Rab5 (early) and Rab11 (recycling) compartments, but has no effect on Ago2 colocalisation with Rab7 (late endosomes).
Unfortunately, we were unable to define a suitable PICK1 mutation that selectively disrupted Ago2 binding without interfering with other important properties of the PICK1 C-terminal tail.
As an alternative approach, we aimed to disrupt endogenous PICK1-Ago2 interactions using a competing peptide. We show in figure 1E that the C-tail of PICK1, (Δ354) is sufficient to bind Ago2. In an attempt to narrow down the Ago2 binding site on PICK1, we made smaller fragments of the isolated C-tail. None of these fragments supported an interaction with Ago2, indicating that the intact C-tail is the minimal Ago2 binding region. Hence we used PICK1 Δ354 to compete with endogenous PICK1 for binding to Ago2 in neurons. In these experiments, we show that disrupting the PICK1-Ago2 interaction causes a reduction in colocalisation with Rab11 compartments, and has no effect on colocalisation with Rab5 or Rab7.
These results support a model in which PICK1 mediates the association of Ago2 with recycling endosomes.
PICK1 shRNA causes a significant reduction in Ago2 colocalisation with Rab5 and Rab11, while PICK1 Δ354 only affects the association with Rab11 compartments. This discrepancy might be explained by the fact that our shRNA causes a 98% reduction in PICK1 expression (Supplementary Figure 1A), while PICK1 Δ354 relies on competition with endogenous PICK1. Therefore in PICK1 shRNA expressing neurons, a negligible proportion of Ago2 is bound to PICK1, whereas PICK1 Δ354-expressing neurons may exhibit some residual Ago2-PICK1 interactions that maintain an association with early endosomes. Moreover, there may be additional as yet unidentified factors that also influence Ago2 localisation to specific endosomal compartments, which could account for an apparent bias for Rab5 or Rab11 compartments.
The other known interactor with the PICK1 C-tail is the Arp2/3 complex. However, it is important to highlight the fact that Δ354 is not sufficient to support Arp2/3 binding (Rocca et al., 2008). Hence PICK1-Arp2/3 interactions would be unaffected by expression of this fragment.

2. Equally important is the demonstration that complex formation between Ago2 and PICK1 indeed does regulate local translation (i.e. local amounts) of endogenous neuronal proteins.

Our experiments using the *limk* 3' UTR - luciferase reporter constructs suggest that PICK1 could regulate the translational repression of endogenous Lim kinase (Limk1). To test this, we analysed endogenous LimK1 expression in neurons with reduced PICK1 by immunocytochemistry. In agreement with the luciferase reporter experiments, PICK1 knockdown causes a significant reduction in endogenous LimK1 expression in dendrites.

3. The data shown in fig. 1 appear somewhat preliminary. For example, in fig. 1A it Ago2 also binds to GST-SNX1 (albeit perhaps more weakly); figs. 1B + 1C miss essential positive (i.e. the antigen itself) and negative controls. Moreover, it is difficult to judge the efficiency of the co-ips and pulldowns as no information is provided what fraction of the input material was loaded for comparison. Fig. 1D misses an input control altogether.

Figure 1A: Given the much larger amount of control GST and GST-SNX1 compared to GST-PICK1 bound to beads (GST blot, bottom panel), the experiment presented indicates that the interaction between Ago2 and GST-PICK1 is dramatically stronger than that of

GST-SNX1, and that a negligible amount of Ago2 is bound to control GST. We therefore feel that it is reasonable to conclude a specific interaction between GST-PICK1 and Ago2. In the revised manuscript, we have annotated the blots to state the fraction of the input loaded, and replaced Fig1D for a blot that includes an input control.

4. I am also unsatisfied with the quantification of colocalizations in Fig. 2. Normalized Pearson coefficents are hard to interpret and a much more extensive analysis with markers for LEs, MVBs, P-bodies etc. is required (see point #1 above).

In the revised manuscript, we have carried out Mander's analysis for Ago2 colocalisation with Rab11, EEA1 and Dcp1a in the absence or presence of PICK1. PICK1 co-expression causes a two-fold increase in the colocalisation of Ago2 with Rab11, but has no effect on Ago2-EEA1 or Ago2-Dcp1 colocalisation. This is consistent with our model that PICK1 regulates the association of Ago2 with recycling endosomes. We have not extended our COS cells analysis to late endosomes, because we investigate this in detail in neurons, as described in point 1, above (in Fig 3 in the revised manuscript).

5. The effect of KD of PICK1 on Ago2 localization to Tf-positive endosomes appears miniscule. Is this because of redundancy in the system? If so, why then does KD of PICK1 exhibit such profound effects on luciferase expression in the reporter assays? From the data it is possible that the effects of PICK1 on gene silencing are mediated by factors other than Ago2. Again, better and more direct data involving interaction-defective mutants, at least in dominant-negative assays are required to substantiate the hypothesis that PICK1 may regulate local translation via Ago2 sequestration.

Our data show that PICK1 knockdown causes a ~24% decrease in Ago2 association with endosomes, and the same treatment causes a ~24-25% decrease in luciferase reporter expression. We feel that these results seem entirely consistent with our model, and do not implicate any factors other than Ago2 in mediating the effects of PICK1 on gene silencing.

Further: Does KD of PICK1 indeed increase the localization of AGo2 with MVBs and P-bodies?
Neither PICK1 knockdown, Δ354PICK1 nor LTD effects Ago2 localisation with the late endosome marker Rab7. These observations suggest that our hypothesis that Ago2 translocates from recycling to late endosomes in response to LTD induction following dissociation from PICK1 was not correct. We have therefore removed this from the revised manuscript. Following dissociation from PICK1, presumably Ago2 translocates to another subcellular compartment where it mediates translational repression via miRNA activity.

<u>Specific responses to Reviewer 2:</u>

1. The authors very often draw conclusions that are not supported by their data. For example, on page 4 (end of first results point) they end with '...suggesting that the pool of Ago2 associated with PICK1 is inactive.' This is not at all demonstrated in Figure 1 or 2. The observation that it does not co-localize with P bodies does not mean that Ago2 is in an inactive form. In fact, the role of P bodies in miRNA-guided gene silencing is rather elusive. This statement should be changed.

We thank the reviewer for pointing this out, and in the revised manuscript we have removed this statement.

2. In the title, the authors claim that PICK1 regulates microRNA processing? I did not find a single experiment in this manuscript that would have tested this hypothesis.

© European Molecular Biology Organization

We apologise for the inaccurate title, which we have now changed.

3. The authors use shRNA-mediated knock down of PICK1. They should also present western blots showing knock down efficiency in their experiments. Especially neurons might be difficult to target and knock down efficiency should be validated.

The shRNA and rescue constructs that we use are identical to the ones used previously by (Citri et al., 2010). The constructs were fully validated in this report, but nevertheless, we have carried out our own characterization here. Supplementary Figure S1A shows that PICK1 shRNA causes a ~98% knockdown of PICK1 expression in neuronal dendrites analysed by immunofluorescence.

4. In Figure 4B, the authors indicate by arrows Ago2-PICK1 co-localization upon treatment with different agents. They claim that LTD inhibits co-localization. Nevertheless, the images look quite similar to the others and also a white arrow indicates co-localization? This should be made clearer.

We acknowledge that in a 20 µm length of dendrite, the subtle reduction in colocalisation may not be obvious to the eye. However, the numerical data and the statistical analysis support the conclusion that LTD causes a ~24% reduction in Ago2-PICK1 colocalisation. In the revised manuscript, we have included an analysis of Ago2 colocalisation with specific endosomal compartments, and we show that LTD causes a specific reduction in colocalisation with Rab11-positive recycling endosomes, with no effect on Rab5 (early) or Rab7 (late) endosomes. These data are presented in Figure 4E, and are consistent with the observed reduction in Ago2-PICK1 colocalisation presented in Figure 4C (previously 4B).

5. Figure 2D is not mentioned in the text.

We apologise for this omission, which has now been rectified.

6. A potential repressive effect of PICK1 on miRNA-guided gene silencing should be tested in more detail. Other proteins should be knocked down as additional controls since the effects are generally quite mild (this holds true for many of the presented experiments, but this might be due to the highly complex neuronal system). The effects on gene silencing are rescued by wt PICK1. An Ago2-binding deficient mutant should not rescue the phenotype. This could be tested. Does PICK1 binding prevent Ago2-GW182 interaction?

Regarding the effect of PICK1 on gene silencing, we note that this comment is at odds with that of Referee 1, who asks, "*why then does KD of PICK1 exhibit such profound effects on luciferase expression in the reporter assays?*" Furthermore, high profile papers using the same *limk1* luciferase reporter constructs report quantitatively similar changes in luciferase activity following a variety of manipulations in neurons, (eg, Schratt et al., 2006). Therefore, we do not feel that further controls are necessary.
Unfortunately, we were unable to define a suitable PICK1 mutation that selectively disrupted Ago2 binding without interfering with other important properties of the PICK1 C-terminus.
As an alternative approach, we aimed to disrupt endogenous PICK1-Ago2 interactions using a competing peptide. We show in figure 1E that the C-terminal tail of PICK1, residues 354-416 are sufficient to bind Ago2. In an attempt to narrow down the Ago2 binding site on PICK1, we made smaller fragments of the isolated C-tail. None of these fragments supported an interaction with Ago2, indicating that the entire C-tail is the minimal Ago2 binding region. Hence we used Δ354PICK1 to compete with endogenous PICK1 for binding to Ago2 in neurons and analysed repression of *limk1* expression, by immunofluorescence of endogenous protein levels and by luciferase reporter assays.

© European Molecular Biology Organization

Δ354PICK1 had no significant effect on expression of either the reporter constructs or of endogenous Limk1. This discrepancy may at first appear to be at odds with our proposed model, however a likely explanation is that the binding site for PICK1 overlaps with other essential Ago2 binding partners. Δ354 PICK1 could therefore inhibit Ago2 function. Hence effects via blocking the PICK1 interaction, which would be expected to enhance Ago2 activity, might be cancelled out by effects on other binding partners. For example, as the reviewer suggests, GW182/TNRC6 proteins bind Ago2 via the PIWI domain and are essential for Ago2 function (Lian et al., 2009; Miyoshi et al., 2009). Indeed, competition with these proteins could be part of the mechanism for the inhibition of Ago2 function by PICK1.

7. Ago-association with membranes has also been analyzed in a recent publication by Stalder et al. (EMBO Journal). This manuscript could be cited as well.

We have referenced this paper in the revised version of the manuscript.

Specific responses to Reviewer 3:

1. In Fig. 1a the authors show blots of AGO2 co-purified with GST-tagged BAR domain proteins. Weak bands for AGO2 are visible in control lanes and SNX1 lanes. The authors should temper there conclusions on the specificity of PICK1 accordingly.

Given the much larger amount of control GST and GST-SNX1 compared to GST-PICK1 bound to beads (GST blot, bottom panel), the experiment presented indicates that the interaction between Ago2 and GST-PICK1 is dramatically stronger than that of GST-SNX1, and that a negligible amount of Ago2 is bound to control GST. We therefore feel that it is reasonable to conclude a specific interaction between GST-PICK1 and Ago2.

2. Fig. 1b several controls are missing for the authors to conclude that PICK1 interacts with AGO2 but not AGO1. First the authors need to demonstrate that they immunoprecipitated equivalent amounts of Flag-PICK1 in each lane. They also need to demonstrate that equivalent amounts of myc-AGO1 and myc-AGO2 were expressed in cells.

Given the referee's concerns, we repeated this experiment, this time using GFP-PICK1 and GFP-trap precipitations. With appropriate expression levels of both myc-Ago1 and myc-Ago2, we found an indistinguishable interaction between PICK1 and Ago1/Ago2. This is the more expected result, since Ago1 and Ago2 are highly homologous in their PIWI domains. The subsequent focus on Ago2 is still justified because it is the best-characterised and is also thought to be the most important Argonaute protein in mammalian RISC (eg, Petri et al., 2011).

3. (a) The authors could build much more confidence in their experiments and reagents by confirming their specificity. This is particularly important since the authors switch between measuring Ago2 localization to Rab11, Tfn and PICK1 in different experiments. For example, the authors should demonstrate that overexpressed PICK1 localizes similarly to endogenous and should highlight data demonstrating that PICK1 labels endosomes (Tfn) and Rab11+ compartments by co-labeling. The authors should similarly confirm PICK1 antibody staining of endosomes.

Regarding the switch between measuring Ago2 localization to Rab11, Tfn and PICK1 in different experiments: our analysis of Ago2 colocalisation with PICK1 is aimed at investigating the conditions for the Ago2-PICK1 interaction, rather than investigating the association of Ago2 with endosomes, which is the aim of the Rab11 or Tfn experiments. Hence we are not using all three interchangeably. However, we acknowledge the referee's concerns about Rab11 and Tfn, and we have now carried out a thorough analysis of Ago2 colocalisation with endosomal compartments defined by the immunolocalisation of specific

Rab proteins. We use Rab5, Rab11 and Rab7 antibodies to label early, recycling and late endosomes respectively.
To show that overexpressed PICK1 has a similar localisation to the endogenous PICK1, we have carried out immunostaining experiments on neurons expressing just endogenous levels of PICK1 and neurons overexpressing GFP-PICK1. In both cases, PICK1 localises to spines, and also to intracellular clusters. This is shown in Supplementary Fig S1 in the revised manuscript. It has been shown before that PICK1 localises to endosomes (Sossa et al., 2006; Madsen et al., 2008). This has been made clear in the revised version of the manuscript (Introduction, p.3; Results p.4).

(b) Staining pattern in Fig. 2A for Rab11 is unusual for recycling endosomes. To confirm the antibody is specific and these are putative recycling endosomes they should treat cells with Rab11A and B-specific siRNA and stain with anti-Rab11 antibody. The authors should show to what extent Rab11 and Tfn foci overlap as well since they often use Tfn as a surrogate marker of Rab11+ or PICK1-affected endosomes. Given that the authors rely on transferrin heavily in the rest of the paper to study AGO2 co-localization with endosomes they should repeat the experiments in Fig. 2A testing AGO2 co-localization with Tfn.

We have now replaced the Rab11 image in Fig 2A for an alternative one, which is more representative of recycling endosome staining.
We use Tfn in initial experiments as a marker for the recycling endosomal system, which would include Rab5 and Rab11 positive compartments, amongst others. In the revised manuscript, we extend the colocalisation analysis to specific endosomal compartments identified by Rab5 (early endosomes), Rab11 (recycling endosomes) and Rab7 (late endosomes) for the key experiments in neurons. Hence in the revised manuscript, we do not rely on Tfn as an endosomal marker. We feel that these additional experiments strongly support our hypothesis of a role for PICK1 in regulating the association of Ago2 with recycling endosomes.

4. For all experiments demonstrating quantification of imaging the authors should note in the figure legend or on the figure itself the number of cells or images analyzed, as well as an average Pearson's correlation coefficient for each condition. It would be helpful to readers to list on the y-axis which two markers were tested for co-localization.

We thank the reviewer for this suggestion. In the revised manuscript, we have included the n-numbers and y-axis labels as suggested. We have now replaced Pearson's for Mander's coefficients for colocalisation, which depend heavily on staining intensity (and therefore on the quality of the antibodies) and also on thresholding used to remove background fluorescence. Therefore, we feel that *absolute* levels of colocalisation do not represent meaningful data.

5. On p.4 the authors state that since PICK1 co-localizes with AGO2 outside P-bodies, that this AGO2 must be in an inactive form. Literature demonstrates that miRNA function normally in the absence of P-bodies. (e.g. P-Body Formation Is a Consequence, Not the Cause, of RNA-Mediated Gene Silencing, Eulalio et al. 2007), therefore the authors' statement should be modified.

We thank the reviewer for pointing this out, and we have removed this claim from the revised manuscript.

6. (a) In FRAP experiments in Fig. 2C-D the authors propose that they examine AGO2 recovery after photobleaching of a foci that overlaps with an Tfn+/PICK1+ foci (presumably an endosome). These experiments require complex controls. First, endosomes are highly dynamic so it is very unlikely that the Tfn+/PICK1+ structure remains stationary in the imaged region if it is an endosome in a healthy cell. Second, the z-plane of capture tends to drift in these experiments, so apparent differences in recovery are easily skewed by

© European Molecular Biology Organization

planes. The authors should show images over the FRAP period with a second marker of the GFP-AGO2 foci that is unaffected by the bleach and demonstrates that the foci is stationary (e.g. Tfn-Alexa647 or mCherry-PICK1 should be more photostable than GFP).

In the revised manuscript, we have included images of cells before and after the time course of the experiment, with all fluorescent markers, to demonstrate the stability of the system. These are presented in Fig 2C in the revised manuscript. As the reviewer suggests, we do observe some movement of clusters, but we only included in our analysis foci that showed little movement for the duration of the recording. We were still able to analyse foci that showed small amounts of movement, provided they remained intact (as defined by the Tfn and PICK1 signals).

(b) The authors cannot conclude from their FRAP experiment that PICK1 stabilizes AGO2 at endosomes. They can only conclude that PICK1 slows recruitment of AGO2 to endosomes.

FRAP represents the exchange of bleached GFP and fluorescent GFP within a region of interest. The data shown in Fig 2C - E indicate that addition of PICK1 reduces the mobile fraction of Ago2 on endosomes. Hence it reduces the exchange of Ago2 on and off endosomes. In conjunction with our data in figure 3B - E, showing that PICK1 increases Ago2 association with endosomes, the best explanation of the FRAP result is that PICK1 stabilises the association of Ago2 with endosomes.

7. The authors frequently test the function of PICK1 by overexpression. The authors need to show western blots of endogenous PICK1 levels in cells vs. levels in cells with overexpressed PICK1. Similarly the authors need to show evidence by western blot of knockdown of PICK1 in Fig. 3-4 and rescue with PICK1 and mutated PICK1.

The shRNA and rescue constructs that we use are identical to the ones used previously by (Citri et al., 2010). The constructs were fully validated in this report, but nevertheless, we have carried out our own characterization here using immunocytochemistry. Supplementary Fig 1A demonstrates that the PICK1 shRNA causes a ~98% reduction in PICK1 expression in transfected neurons. The level of PICK1 overexpression in neurons is quantified in Supplementary Fig S1B. PICK1 is not expressed at significant levels in COS7 cells.

8.Given that many of the effects the authors observe are weak the statistical tests they applied are important. The authors should stipulate whether one- or two-tailed t-tests were applied, the type, and the p-value considered significant. At times ANOVA tests may be required to compare more than two samples.

We apologise for omitting details of the statistical tests. In the revised manuscript, we have included all p-values, and stated which test was used. In all cases, two-tailed tests were used. We acknowledge that in some cases, a post-hoc correction for multiple comparisons may be appropriate. Such a correction would alter the threshold for significance. However, in the cases where this might apply, our p-values are sufficiently low to be unaffected by such a correction.

9. The functional or physiological relevance of the paper could be significantly increased if the authors reinforced the data showing an effect on miRNA activity (NOT processing) by testing whether PICK1 affects levels of LIMK1 protein in neurons and affects dendritic spine morphology as suggested by the miR-134 vector that they use from Schratt GM et al. Nature 2006.

Our experiments using the *limk* 3' UTR - luciferase reporter constructs suggest that PICK1 could regulate the translational repression of endogenous Lim kinase (Limk1). To test this,

we analysed endogenous LimK1 expression in neurons with reduced PICK1 by immunocytochemistry. In agreement with the luciferase reporter experiments, PICK1 knockdown causes a significant reduction in LimK1 expression in dendrites, demonstrating that PICK1 modulates the expression of endogenous Lim kinase.
It is already known that PICK1 regulates dendritic spine size (Nakamura et al., 2011), an effect that was shown to be via direct modulation of the actin-nucleating Arp2/3 complex.

10. I would recommend changing the title from "MiRNA Processing" to "MiRNA Activity". MiRNA processing suggests that the observed effects are on pre-miRNA cleavage or other stages of miRNA biogenesis or modification.

We apologise for the inaccurate title, which we have now changed.

MINOR POINTS

1. In Fig. 1b the IP is labeled as myc-AGO1 or -2 and the blot is labeled as myc-AGO. One of these is mislabeled.

We thank the reviewer for pointing this out. Figure 1b has been replaced, and has been labeled correctly in the revised manuscript.

2. The IP-western blot in Fig. 4a is not labeled with the identities of the proteins blotted.
We thank the reviewer for pointing out this omission, which we have now rectified.

3. Fig. 1E would suggest that the BAR domain inhibits binding of AGO2 to the PICK1 C-terminus. What might this imply for localization at endosomes because of known functions of the BAR domain? What if anything is known about the AGO2 binding site in the PICK1 C-terminus? The authors should comment.

This is an interesting point. As the reviewer suggests, the BAR domain could inhibit the interaction with Ago2. Since PICK1 associates with endosomes via the BAR domain, it might suggest that PICK1 association with endosomes would strengthen the Ago2-PICK1 interaction. This would further support our model that PICK1 increases Ago2 association with endosomes.
We carried out experiments with the aim of further defining the Ago2 binding site on PICK1. However, we found that smaller fragments of the PICK1 CT did not support Ago2 binding, indicating that the entire 354-416 region is required for the interaction.

<u>References</u>

Citri, A., Bhattacharyya, S., Ma, C., Morishita, W., Fang, S., Rizo, J., and Malenka, R.C. (2010). Calcium binding to PICK1 is essential for the intracellular retention of AMPA receptors underlying long-term depression. The Journal of neuroscience : the official journal of the Society for Neuroscience 30, 16437-16452.

Lian, S.L., Li, S., Abadal, G.X., Pauley, B.A., Fritzler, M.J., and Chan, E.K. (2009). The C-terminal half of human Ago2 binds to multiple GW-rich regions of GW182 and requires GW182 to mediate silencing. Rna 15, 804-813.

Madsen, K.L., Eriksen, J., Milan-Lobo, L., Han, D.S., Niv, M.Y., Ammendrup-Johnsen, I., Henriksen, U., Bhatia, V.K., Stamou, D., Sitte, H.H.*, et al.* (2008). Membrane localization is critical for activation of the PICK1 BAR domain. Traffic 9, 1327-1343.

Miyoshi, K., Okada, T.N., Siomi, H., and Siomi, M.C. (2009). Characterization of the miRNA-RISC loading complex and miRNA-RISC formed in the Drosophila miRNA pathway. Rna 15, 1282-1291.

© European Molecular Biology Organization

Nakamura, Y., Wood, C.L., Patton, A.P., Jaafari, N., Henley, J.M., Mellor, J.R., and Hanley, J.G. (2011). PICK1 inhibition of the Arp2/3 complex controls dendritic spine size and synaptic plasticity. The EMBO journal 30, 719-730.

Petri, S., Dueck, A., Lehmann, G., Putz, N., Rudel, S., Kremmer, E., and Meister, G. (2011). Increased siRNA duplex stability correlates with reduced off-target and elevated on-target effects. Rna 17, 737-749.

Rocca, D.L., Martin, S., Jenkins, E.L., and Hanley, J.G. (2008). Inhibition of Arp2/3-mediated actin polymerization by PICK1 regulates neuronal morphology and AMPA receptor endocytosis. Nat Cell Biol 10, 259-271.

Schratt, G.M., Tuebing, F., Nigh, E.A., Kane, C.G., Sabatini, M.E., Kiebler, M., and Greenberg, M.E. (2006). A brain-specific microRNA regulates dendritic spine development. Nature 439, 283-289.

Sossa, K.G., Court, B.L., and Carroll, R.C. (2006). NMDA receptors mediate calcium-dependent, bidirectional changes in dendritic PICK1 clustering. Mol Cell Neurosci 31, 574-585.

2nd Editorial Decision 10 January 2014

Many thanks for the submission of your revised study to EMBO reports. The manuscript was sent back to the three original referees and while they overall appreciate that the study has been strengthened during revision, both referee 1 and 3 still raise concerns that would need to be addressed before publication.

For example, referee 1 still maintains that a rescue experiment with a PICK1 mutant that cannot bind to Ago2 should be performed instead of (or in addition to) the dominant-negative approach currently used. This would also eliminate the concern raised by this reviewer with regard to the fact that the delta145 fragment of PICK1 seems unable to bind to Ago2, which could have consequences for the interpretation of the DN experiment. Referee 3 feels that in some instances, the interpretations should be toned down (his/her point 1) and that alternative potential interpretations should at least be discussed (note this referees' comment no. 2). This reviewer also maintains that the quantification of the co-localization data of Ago2 at the endosomes needs to be strengthened (point 3) and that the statistical tests still need to be improved (comment 4). Finally, s/he states that a control for the knockdown efficiency of PICK1 is needed.

Given the overall interest the referees expressed in your study, I would like to give you the exceptional opportunity to revise the study a second time, with the understanding that the concerns of referee 1 and 3 concerns have to be addressed before the study can be published in EMBO reports. When submitting the final version of your manuscript, please also outline briefly in a point-by-point manner how you have addressed the remaining referee concerns.

Since the total time since the initial submission is already more than six months, I would kindly ask you to submit the revised study within about four weeks.

Please do let me know if you anticipate problems with this time-frame, as I am sure we can find a solution.

REFEREE REPORTS:

Referee #1:

The revised Ms is improved over the previous version though a few issues remain that need to be

© European Molecular Biology Organization

solved prior to publication:

1. The authors now use a DN approach emloying a fragment of PICK1 to address the question of whether really complex formation between Ago2 and PICK1 underlies the observed phenotypes with respect to Ago2 localization and Limk1 expression. While it seems from the previously published work by Hanley and colleagues that indeed delta354 is insufficient for binding it cannot be concluded that the observed effects with respect to Ago2 localization are truly reflecting complex formation rather than indirect effects caused by other binding partners (i.e. endogenous PICK1; see point #2 below).
A much more straightforward experiment would be to test if mutant PICK1 lacking the C-terminal Ago2 binding tail is able to rescue loss of PICK1 with respect to Ago2 targeting to Rab11 endosomes and Limk1 expression. I see no obvious reason why this epxeriment has not been conducted (as suggested in the initial review).

2. I am puzzled by the fact that delta354 in fig. 1E binds Ago2 comparably well whereas delta145 is hardly above the GST background - yet, similar amounts of both GST-tagged proteins appear to have been offered. Is PICK1 autoinhibited? This could be of interest with respect to the mechanism of cLTD and release of Ago2 and might cloud the interpretation of DN experiments (see #1 above) as delta145 could conceivably alter the activity of endogenous PICK1, for example by occluding its BAR domain.

3. The effect of cLTD on Ago2 localization to Rab11 endosomes in fig. 4E is miniscule. The authors thus should downtone their corresponding statement in the text.

4. Minor: In the main Ms text it is stated that co-ips in Fig. 1B were done from Hek293 cells while the legends refers to Cos7. Which is true? The ctrl is not described in the legend- what is the sample?

Referee #2:

In the revised version of their manuscript, the authors have addressed the points that I had raised on their previous version. I am satisfied with the comments and the revised manuscript.

Referee #3:

The manuscript has improved significantly from the previous submission. In principle, the message that PICK1 affects dendritic remodeling at least in part by affecting miRNA activity is interesting and reasonably supported. My main problem at present with the manuscript is that the authors push too hard for certain interpretations that are at best only partly supported by the evidence. The authors focus on endosomes. This is understandable given the literature on Ago2 and endosomes, including the recent Mol. Cell paper from Richard Carthew's lab. However, PICK1 localizes to endosomes only weakly and affects actin dynamics and likely other trafficking pathways. Indeed, PICK1 has also been shown to localize to mitochondria, Golgi and affect secretory vesicle biogenesis. Therefore, the authors may observe a small effect on Ago localization to endosomes, but this might be an indirect consequence of other PICK1 functions, and not due to PICK1 directly interacting with Ago2 on endosomes. I can accept that the authors show an association (possibly indirect) of Pick1 with Ago2, and that this affects Ago2 localization to endosomes in a weak way, but given the poor overlap of staining of PICK1 with endosome markers the authors statements that PICK1 binds Ago2 on endosomes is unsupported. This is one example among several in the cell biology section of the paper where the authors need to be much more cautious in their interpretations.
1. The authors interpretation of FRAP experiments is very speculative. FRAP only measures increases in fluorescence at the bleached site. It gives no indication of what happens to the bleached molecules, GFP-Ago2 in their case. Therefore the authors only know from their experiment that GFP-Ago2 recruitment to endosomes is slowed, they know nothing about the GFP-Ago2 that was already on endosomes. Their claim that these FRAP experiments demonstrate that PICK1 stabilizes GFP-Ago2 on endosomes is based on several assumptions: that a finite number of binding sites are present on endosomes for GFP-Ago2 that are saturated at the beginning of the experiment and that

recruitment of new GFP-Ago2 to endosomes would only be possible if some of these finite binding sites are liberated by departure of GFP-Ago2.In light of the authors data (albeit weak) that PICK1 increases colocalization of Ago2 with Rab11 endosomes, one interpretation among other possible interpretations would be that PICK1 stabilizes Ago2 on recycling endosomes, but this is not demonstrated by FRAP alone, and is more hypothesis than solid evidence.
2. The authors results demonstrate that Ago associates with PICK1, and this interaction requires the PICK1 C-terminal domain, which also allows PICK1 to associate with membranes and endosomes. An alternative explanation of the authors results would be that in immunoprecipitating PICK1 large membrane fragments of endosomes are pulled-down, which contain Ago2 among other endosome-associated proteins. Do the authors have evidence that would disprove this possibility and suggest a direct interaction of PICK1 with Ago2?
3. The co-localization the authors observe between Ago2 and endosomes is weak to the eye. In point 4 I requested the authors show the average Pearson's correlation coefficient for each co-localization the authors quantitatively evaluated. This was requested because this gives readers a quantitative idea of the significance of any colocalization in these experiments. These values have not been provided. If the authors want to rely on Mander's coefficients they should provide an average of these correlation coefficients for each co-localization quantitatively evaluated. Ideally, the authors would test the significance of colocalization using Costes' or Fayes' methods. As well, two Mander's values are generated for each co-localization, one measuring colocalization of A with B and the other of B with A. The authors should be clear which one they are using and justify not using both. These are highly complex analyses that are very error prone if not elaborately controlled.The authors need to moderate their claims based on use of these co-localization analyses.
4. In point 6 I had asked the authors to perform appropriate statistical tests for the samples analyzed. While I appreciate their assurances that this wouldn't change the significance of their findings I still think they should use the appropriate statistical tests.
5. In point 7 I asked the authors to provide evidence that the PICK1 shRNA they use efficiently reduces PICK1 levels. While the authors refer to this evidence being in Supp. Fig. 1A I cannot find this evidence in this figure. The only evidence I see is that over-expression of GFP-PICK1 increases the immunofluorescence signal for PICK1. I do not consider evidence that another group has successfully used this shRNA construct as proof of knockdown. In most circumstances western blot or at minimum RT-qPCR would be required to test this. According to Citri et al. and what I understand of the authors discussion of the PICK1 shRNA plasmid, it co-expresses GFP to identify transfected cells. The authors should be able to test knockdown in neurons by western blot after flow cytometry sorting or other methods using this GFP as Citri et al. did. This however raises another important concern. How do the authors confidently differentiate signals that come from GFP expressed by the shRNA plasmid from signals due to GFP-PICK1 in the series of rescue experiments they perform (Fig. 3B-E, Supp. Fig. 1A, Fig. 4A)? The expression of GFP by these two plasmids would make it impossible to distinguish free GFP from GFP-PICK1 to examine PICK1 co-localization and make it difficult to confidently identify which cells express the shRNA alone, shRNA and GFP-PICK1, or GFP-PICK1 alone to select cells for quantification of immunofluorescence. This would put in doubt many of the results in Fig. 3B-E, Fig. 4A and Supp. Fig.1A.

2nd Revision - authors' response 03 February 2014

Response to reviewers

We thank the reviewers for their constructive comments, which we have addressed point-by-point, below.

Specific responses to Reviewer 1:

1. The authors now use a DN approach employing a fragment of PICK1 to address the question of whether really complex formation between Ago2 and PICK1 underlies the observed phenotypes with respect to Ago2 localization and Limk1 expression. While it

seems from the previously published work by Hanley and colleagues that indeed delta354 is insufficient for binding it cannot be concluded that the observed effects with respect to Ago2 localization are truly reflecting complex formation rather than indirect effects caused by other binding partners (i.e. endogenous PICK1; see point #2 below).
A much more straightforward experiment would be to test if mutant PICK1 lacking the C-terminal Ago2 binding tail is able to rescue loss of PICK1 with respect to Ago2 targeting to Rab11 endosomes and Limk1 expression. I see no obvious reason why this experiment has not been conducted (as suggested in the initial review).

Following the comments from the first review, our aim was to identify a suitable mutant that could be used in such an experiment. As we stated in our response to the first review, unfortunately we were unable to define such a mutant that would not interfere with other functions of PICK1.
We feel that experiments using a PICK1 mutant lacking the C-tail (as the reviewer suggests) would be difficult to interpret because of the multiple effects of deleting this domain. A C-terminal truncation would abolish not only Ago2 binding, but also Arp2/3 binding, which is crucial for LTD mechanisms (Nakamura et al., 2011; Rocca et al., 2008). It would also influence Ca^{2+} binding via the acidic region located in the C-tail (Hanley and Henley, 2005). Ca^{2+} sensing by PICK1 is also critical for LTD (Citri et al., 2010). Furthermore, C-terminal deletion has been suggested to enhance the interaction of PICK1 with lipid membranes (Jin et al., 2006). Since we are investigating the role of PICK1 in modulating the association of Ago2 with endosomal compartments, this will also be an important confounding factor.
Hence, we believe that our approach of using the fragment of PICK1 to compete with endogenous PICK1 for Ago2 binding is the most appropriate.

2. I am puzzled by the fact that delta354 in fig. 1E binds Ago2 comparably well whereas delta145 is hardly above the GST background - yet, similar amounts of both GST-tagged proteins appear to have been offered. Is PICK1 autoinhibited? This could be of interest with respect to the mechanism of cLTD and release of Ago2 and might cloud the interpretation of DN experiments (see #1 above) as delta145 could conceivably alter the activity of endogenous PICK1, for example by occluding its BAR domain.

There is considerable evidence that PICK1 is indeed autoinhibited. It has been shown that the isolated PDZ domain binds directly to the isolated BAR domain (Lu and Ziff, 2005; Rocca et al., 2008). In the intact protein, it is thought that the PDZ domain folds in on the BAR domain, masking certain BAR domain interactions. Occupation of the PDZ domain with a PDZ ligand unmasks this inhibition (Lu and Ziff, 2005). To our knowledge, there is no evidence that the C-terminal tail can bind directly to other regions of PICK1, although it is conceivable that the BAR domain could mask C-tail interactions (not necessarily via direct binding of BAR domain to C-tail), including Ago2. In this hypothetical situation, the PDZ domain might inhibit this property of the BAR domain, hence in the absence of the PDZ domain (Δ145), the BAR domain would be available to inhibit Ago2 binding. Such a model could explain the result in Figure 1E, which suggests that PDZ deletion reduces the interaction with Ago2.
Furthermore, during LTD, PICK1 binds AMPA receptor subunit GluA2. As mentioned above, PDZ domain occupation can have a similar effect on BAR domain availability as PDZ domain deletion. Hence, during LTD, this could represent a mechanism for the observed reduction in PICK1-Ago2 binding, as alluded to by the reviewer.
The referee refers to Δ145 altering the activity of the BAR domain of endogenous PICK1. We assume the referee means Δ354. While we acknowledge that this is a formal possibility, it is important to stress the lack of published evidence to suggest that the isolated C-tail can bind directly to any other region on PICK1.

3. The effect of cLTD on Ago2 localization to Rab11 endosomes in fig. 4E is miniscule. The authors thus should downtone their corresponding statement in the text.

© European Molecular Biology Organization

We have added an additional sentence in the final paragraph (page 8) acknowledging the small proportion of Ago2 that dissociates from recycling endosomes following cLTD induction.

4. Minor: In the main Ms text it is stated that co-ips in Fig. 1B were done from Hek293 cells while the legends refers to Cos7. Which is true? The ctrl is not described in the legend- what is the sample?

We apolgise for this error. These experiments were done in HEK293 cells. This has now been rectified in the revised manuscript.
The control sample is the same lysate as the GFP-trap, but it is incubated with blocked agarose beads, which are treated the same as the GFP-trap agarose beads. We have now included this information in the legend.

<u>Specific responses to Reviewer 3:</u>

1. The authors interpretation of FRAP experiments is very speculative. FRAP only measures increases in fluorescence at the bleached site. It gives no indication of what happens to the bleached molecules, GFP-Ago2 in their case. Therefore the authors only know from their experiment that GFP-Ago2 recruitment to endosomes is slowed, they know nothing about the GFP-Ago2 that was already on endosomes. Their claim that these FRAP experiments demonstrate that PICK1 stabilizes GFP-Ago2 on endosomes is based on several assumptions: that a finite number of binding sites are present on endosomes for GFP-Ago2 that are saturated at the beginning of the experiment and that recruitment of new GFP-Ago2 to endosomes would only be possible if some of these finite binding sites are liberated by departure of GFP-Ago2.In light of the authors data (albeit weak) that PICK1 increases colocalization of Ago2 with Rab11 endosomes, one interpretation among other possible interpretations would be that PICK1 stabilizes Ago2 on recycling endosomes, but this is not demonstrated by FRAP alone, and is more hypothesis than solid evidence.

Non-covalent protein interactions involve a dynamic equilibrium of binding and unbinding. Hence in our system, Ago2 continually dissociates from endosomes and re-associates. We therefore assume that following bleaching, a proportion of bleached GFP-Ago2 moves out of the region of interest (ROI), and fluorescent GFP-Ago2 moves into the ROI.
If the recovery was purely due to recruitment of new GFP-Ago2 to endosomes, with no simultaneous unbinding of existing (bleached) GFP-Ago2, this would imply a one-directional flow of GFP-Ago2 towards endosomes, which would be unsustainable.
Although we acknowledge that the number of Ago2 binding sites on endosomes might be variable, we see no reason why photobleaching would change the number of Ago2 binding sites on endosomes. Hence we do assume that during the course of the experiment (~4 min), the number of binding sites is constant, and that recovery of fluorescence involves not only an increase in unbleached GFP-Ago2 in the ROI, but also a loss of bleached GFP-Ago2.
Fluorescence recovery is therefore a measure of exchange of molecules between the ROI and the surrounding area. This view is supported by numerous reviews on the subject (for example Gonzalez-Gonzalez et al., 2012; Staras et al., 2013).
However, we acknowledge that our interpretation of the data is not the only possibility, so we have added a statement to the text including the alternative scenario that PICK1 slows Ago2 recruitment to endosomes (page 5).

2. The authors results demonstrate that Ago associates with PICK1, and this interaction requires the PICK1 C-terminal domain, which also allows PICK1 to associate with membranes and endosomes. An alternative explanation of the authors results would be that in immunoprecipitating PICK1 large membrane fragments of endosomes are pulled-down, which contain Ago2 among other endosome-associated proteins. Do the authors

have evidence that would disprove this possibility and suggest a direct interaction of PICK1 with Ago2?

The association of PICK1 with membranes and endosomes is mediated by the BAR domain, with some positive contribution from the PDZ domain (Jin et al., 2006; Madsen et al., 2008; Pan et al., 2007). It has previously been suggested that PICK1 C-tail actually inhibits binding to lipid membranes (Jin et al., 2006). These previous studies do not support the reviewer's suggestion that the PICK1 C-terminal domain allows PICK1 to associate with membranes and endosomes. Therefore, while we acknowledge that our results do not formally prove a direct interaction, we do not believe that GST-Δ354 would interact with large membrane fragments of endosomes. This would also be unlikely in detergent-solubilised extracts. Furthermore, in the proteomics screen that identified Ago2 as a PICK1 interactor, the profile of interacting proteins did not suggest a non-specific association with endosomes.

3. The co-localization the authors observe between Ago2 and endosomes is weak to the eye. In point 4 I requested the authors show the average Pearson's correlation coefficient for each co-localization the authors quantitatively evaluated. This was requested because this gives readers a quantitative idea of the significance of any colocalization in these experiments. These values have not been provided. If the authors want to rely on Mander's coefficients they should provide an average of these correlation coefficients for each co-localization quantitatively evaluated. Ideally, the authors would test the significance of colocalization using Costes' or Fayes' methods. As well, two Mander's values are generated for each co-localization, one measuring colocalization of A with B and the other of B with A. The authors should be clear which one they are using and justify not using both. These are highly complex analyses that are very error prone if not elaborately controlled.The authors
need to moderate their claims based on use of these co-localization analyses.

We acknowledge the reviewer's concerns about the colocalisation analysis. The figure legends already included information about which of the two possible Mander's coefficients we used. In all cases, we determined the fraction of Ago2 that colocalises with the relevant endosomal marker. The reason for using this value is that we are studying the regulation of the pool of Ago2 that colocalises with endosomal markers, and not the pool of endosomal markers that colocalise with Ago2.
The colocalisation data were acquired over a number of independent experiments, each of which was carried out in independent immunocytochemistry sessions. Immunocytochemistry cannot be relied on to be exactly reproducible from one experiment to the next, and the microscope settings for the consequent imaging often need to be adjusted between sessions to account for overall differences in staining intensity or background fluorescence, etc. Therefore the results from one session should not be directly pooled with those of another. By normalizing the data for each session, the variability of the immunocytochemistry is controlled for. Hence averages of the actual Mander's coefficients are not particularly meaningful, because they do not account for this slight variability between sessions.

Nevertheless, we include the Mander's coefficents here:

Fig.2B:

	-PICK1	+PICK1
Ago2-Rab11	0.0474	0.0570
Ago2-EEA1	0.0377	0.0279
Ago2-Dcp1	0.0481	0.0433

© European Molecular Biology Organization

Fig.3B:

	shPICK1+ WT	shPICK1+GFP	shPICK1+ 5K/E
Ago2-Tfn	0.1949	0.1502	0.1377

Fig.3C:

	GFP	GFP-PICK1
Ago2-Tfn	0.1018	0.1625

Fig.3D:

	shPICK1+PICK1	shPICK1+GFP
Ago2-Rab5	0.0895	0.0721
Ago2-Rab11	0.1194	0.0921
Ago2-Rab7	0.1082	0.1231

Fig.3E:

	GFP	delta354
Ago2-Rab5	0.1539	0.1553
Ago2-Rab11	0.1534	0.1240
Ago2-Rab7	0.1221	0.1396

Fig.4C:

control	TTX	LTD	BIC
0.239466095	0.2382	0.1904	0.2605

Fig.4E:

	Control	LTD
Ago2-Rab5	0.1310	0.1326
Ago2-Rab11	0.1188	0.1002
Ago2-Rab7	0.1435	0.1300

4. In point 6 I had asked the authors to perform appropriate statistical tests for the samples analyzed. While I appreciate their assurances that this wouldn't change the significance of their findings I still think they should use the appropriate statistical tests.

We have now applied a Bonferroni correction for multiple comparisons (to avoid type I errors) to the data sets that involve more than one comparison to a single control. These are Figures 3B, 4A, 4B, 4C, 4D.

5. In point 7 I asked the authors to provide evidence that the PICK1 shRNA they use efficiently reduces PICK1 levels. While the authors refer to this evidence being in Supp. Fig. 1A I cannot find this evidence in this figure. The only evidence I see is that over-expression of GFP-PICK1 increases the immunofluorescence signal for PICK1. I do not consider evidence that another group has successfully used this shRNA construct as proof of knockdown. In most circumstances western blot or at minimum RT-qPCR would be required to test this. According to Citri et al. and what I understand of the authors discussion of the PICK1 shRNA plasmid, it co-expresses GFP to identify transfected cells. The authors should be able to test knockdown in neurons by western blot after flow cytometry sorting or other methods using this GFP as Citri et al. did. This however raises another important concern. How do the authors confidently differentiate signals that come from GFP expressed by the shRNA plasmid from signals due to GFP-PICK1 in the series of rescue experiments they perform (Fig. 3B-E, Supp. Fig. 1A, Fig. 4A)? The expression of GFP by these two plasmids would make it impossible to distinguish free GFP from GFP-PICK1 to examine PICK1 co-localization and make it difficult to confidently identify which cells express the shRNA alone, shRNA and GFP-PICK1, or GFP-PICK1 alone to select cells for quantification of immunofluorescence. This would put in doubt many of the results in Fig. 3B-E, Fig. 4A and Supp. Fig.1A.

© European Molecular Biology Organization

We apologise for the omission. The data in Figure S1 A and B were acquired at the same time, in the same set of experiments, hence the same GFP control applies to both. We have therefore combined the graphs in Figure S1 so that the shRNA condition is directly comparable to the control. This clearly shows that PICK1 shRNA causes a dramatic reduction in endogenous PICK1 expression. We hope the revised version clarifies this point.

The rescue experiments employ a single plasmid that expresses shRNA in conjunction with either free GFP or GFP-PICK1. Hence, in all these experiments, there is only a single GFP species present. We apologise for not making this clear, and we have added additional explanation about these constructs to the methods section in the revised manuscript.

References:

Citri, A., Bhattacharyya, S., Ma, C., Morishita, W., Fang, S., Rizo, J., and Malenka, R.C. (2010). Calcium binding to PICK1 is essential for the intracellular retention of AMPA receptors underlying long-term depression. The Journal of neuroscience : the official journal of the Society for Neuroscience 30, 16437-16452.

Gonzalez-Gonzalez, I.M., Jaskolski, F., Goldberg, Y., Ashby, M.C., and Henley, J.M. (2012). Measuring membrane protein dynamics in neurons using fluorescence recovery after photobleach. Methods Enzymol 504, 127-146.

Hanley, J.G., and Henley, J.M. (2005). PICK1 is a calcium-sensor for NMDA-induced AMPA receptor trafficking. EMBO J 24, 3266-3278.

Jin, W., Ge, W.P., Xu, J., Cao, M., Peng, L., Yung, W., Liao, D., Duan, S., Zhang, M., and Xia, J. (2006). Lipid binding regulates synaptic targeting of PICK1, AMPA receptor trafficking, and synaptic plasticity. J Neurosci 26, 2380-2390.

Lu, W., and Ziff, E.B. (2005). PICK1 interacts with ABP/GRIP to regulate AMPA receptor trafficking. Neuron 47, 407-421.

Madsen, K.L., Eriksen, J., Milan-Lobo, L., Han, D.S., Niv, M.Y., Ammendrup-Johnsen, I., Henriksen, U., Bhatia, V.K., Stamou, D., Sitte, H.H., *et al.* (2008). Membrane localization is critical for activation of the PICK1 BAR domain. Traffic 9, 1327-1343.

Nakamura, Y., Wood, C.L., Patton, A.P., Jaafari, N., Henley, J.M., Mellor, J.R., and Hanley, J.G. (2011). PICK1 inhibition of the Arp2/3 complex controls dendritic spine size and synaptic plasticity. The EMBO journal 30, 719-730.

Pan, L., Wu, H., Shen, C., Shi, Y., Jin, W., Xia, J., and Zhang, M. (2007). Clustering and synaptic targeting of PICK1 requires direct interaction between the PDZ domain and lipid membranes. EMBO J 26, 4576-4587.

Rocca, D.L., Martin, S., Jenkins, E.L., and Hanley, J.G. (2008). Inhibition of Arp2/3-mediated actin polymerization by PICK1 regulates neuronal morphology and AMPA receptor endocytosis. Nat Cell Biol 10, 259-271.

Staras, K., Mikulincer, D., and Gitler, D. (2013). Monitoring and quantifying dynamic physiological processes in live neurons using fluorescence recovery after photobleaching. Journal of neurochemistry 126, 213-222.

© European Molecular Biology Organization

3rd Editorial Decision 25 February 2014

I am very happy to tell you that the two referees that were asked to assess the final version of your manuscript now fully support its publication in EMBO reports.

As part of the EMBO publication's Transparent Editorial Process, EMBO reports publishes online a Review Process File to accompany accepted manuscripts. As you are aware, this File will be published in conjunction with your paper and will include the referee reports, your point-by-point response and all pertinent correspondence relating to the manuscript.

If you do NOT want this File to be published, please inform the editorial office within 2 days, if you have not done so already, otherwise the File will be published by default [contact: emboreports@embo.org]. If you do opt out, the Review Process File link will point to the following statement: "No Review Process File is available with this article, as the authors have chosen not to make the review process public in this case."

Thank you again for your contribution to EMBO reports and congratulations on a successful publication. Please consider us again in the future for your most exciting work.

© European Molecular Biology Organization
