# Supplementary figures and images for: PICK1 links Argonaute 2 to endosomes in neuronal dendrites and regulates miRNA activity
Source: EMBO Rep. 2014 Apr 10;15(5):548–56. doi: 10.1002/embr.201337631 (PMC4210090; doi:10.1002/embr.201337631)

shPICK1

PICK1

-

PICK1-5K/E

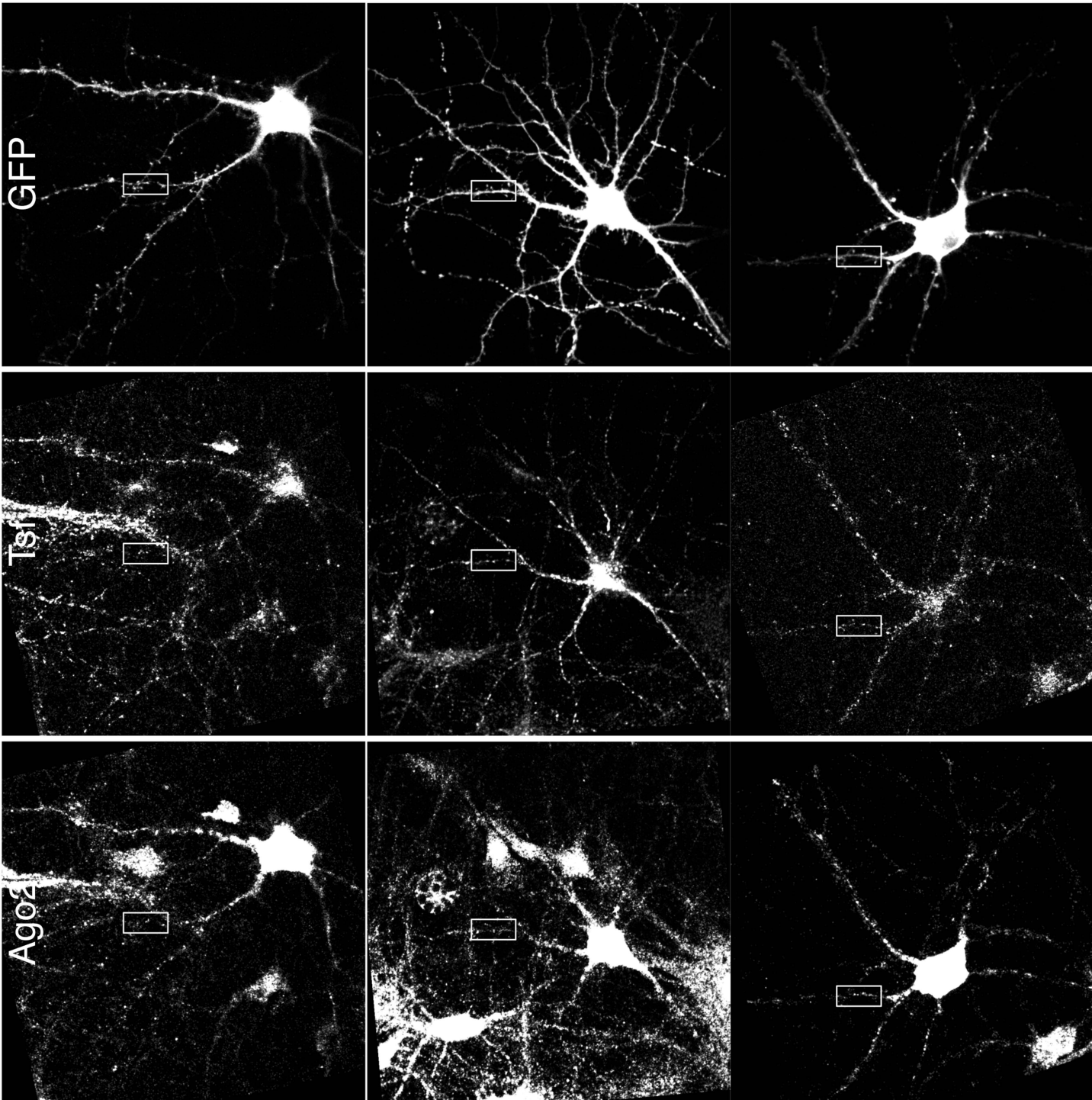

Supplement: Supplementary file 5 [file embr0015-0548-sd5.pdf]

CTRL

PICK1

Ago2

TTX

Bic

LTD

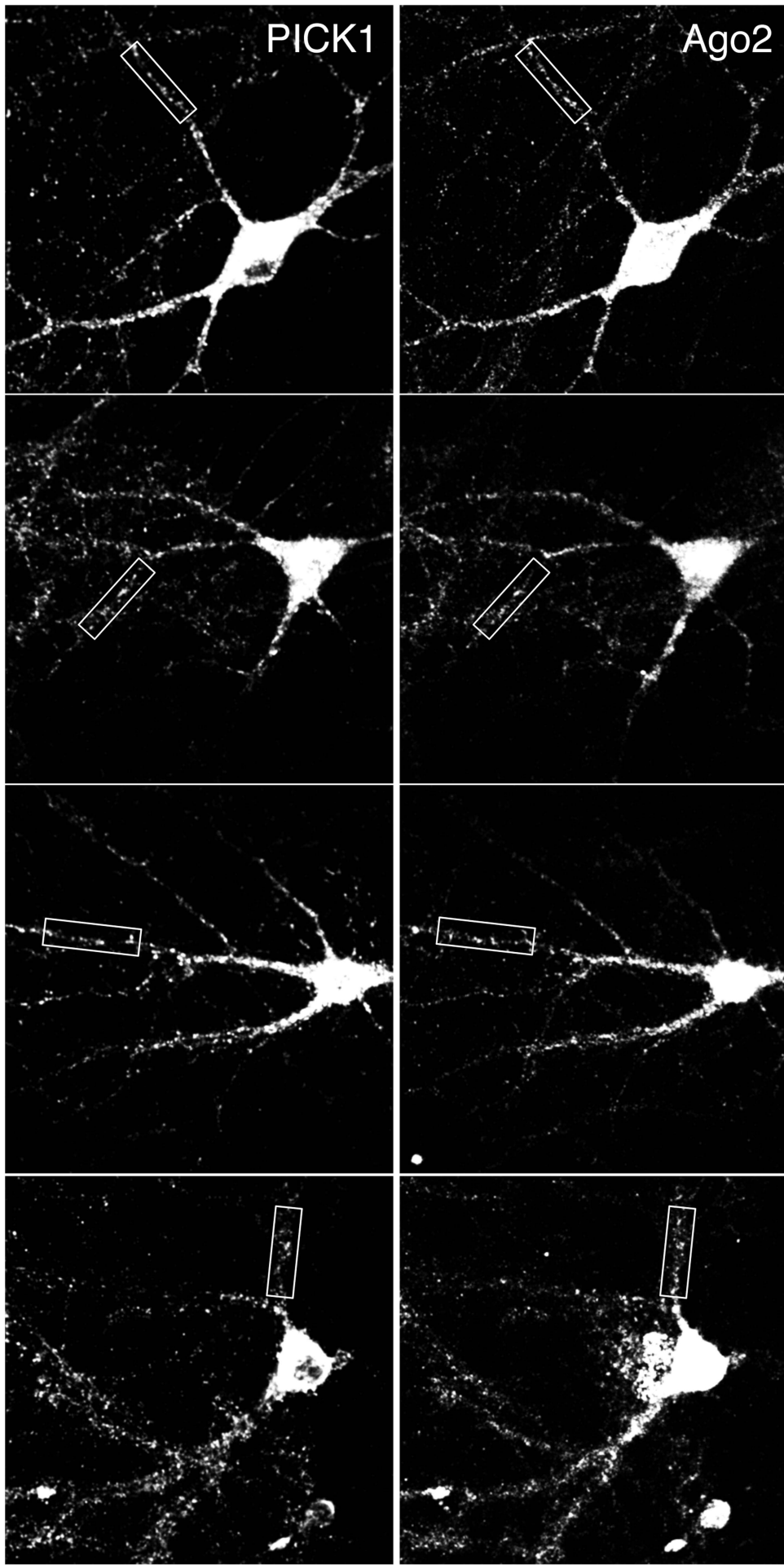

Supplement: Supplementary file 6 [file embr0015-0548-sd6.pdf]
